# A case of antiferrochirality in a liquid crystal phase of counter-rotating staircases

**DOI:** 10.1038/s41467-022-28024-1

**Published:** 2022-01-19

**Authors:** Ya-xin Li, Hong-fei Gao, Rui-bin Zhang, Kutlwano Gabana, Qing Chang, Gillian A. Gehring, Xiao-hong Cheng, Xiang-bing Zeng, Goran Ungar

**Affiliations:** 1 State Key Laboratory for Mechanical Behaviour of Materials, Shaanxi International Research Centre for Soft Matter, Xi’an Jiaotong University, 710049 Xi’an, P. R. China; 2 Department of Materials Science and Engineering, University of Sheffield, Sheffield, S1 3JD UK; 3 Key Laboratory of Medicinal Chemistry from Natural Resources, Ministry of Education, Yunnan University, Kunming, P. R. China; 4 Department of Physics and Astronomy, University of Sheffield, Sheffield, E1 2C UK; 5 School of Chemistry and Chemical Engineering, Yangtze Normal University, Fuling, P. R. China; 6 Present Address: School of Chemistry and Chemical Engineering, Henan University of Technology, 450001 Zhengzhou, P. R. China

**Keywords:** Self-assembly, Liquid crystals

## Abstract

Helical structures continue to inspire, prompted by examples such as DNA double-helix and alpha-helix in proteins. Most synthetic polymers also crystallize as helices, which relieves steric clashes by twisting, while keeping the molecules straight for their ordered packing. In columnar liquid crystals, which often display useful optoelectronic properties, overall helical chirality can be induced by inclusion of chiral chemical groups or dopants; these bias molecular twist to either left or right, analogous to a magnetic field aligning the spins in a paramagnet. In this work, however, we show that liquid-crystalline columns with long-range helical order can form by spontaneous self-assembly of straight- or bent-rod molecules without inclusion of any chiral moiety. A complex lattice with *Fddd* symmetry and 8 columns per unit cell (4 right-, 4 left-handed) characterizes this “antiferrochiral” structure. In selected compounds it allows close packing of their fluorescent groups reducing their bandgap and giving them promising light-emitting properties.

## Abstract (web summary)

A 3D‐ordered liquid crystal phase of regular right and left helically twisted columns self‐assembles from straight‐ and bent‐rod molecules. Here calculations indicate that, among four alternative models, the observed complex *Fddd* structure provides the lowest packing energy for twisted ribbons.

## Introduction

A large part of condensed matter physics has been devoted to the study of the spontaneous long-range orientational ordering of electronic spins, leading to ferromagnetism and antiferromagnetism. Another intriguing self-ordering phenomenon is spontaneous synchronization of chirality, i.e., breaking the mirror symmetry and favoring either right- or left-handedness. A much-studied problem in biology is understanding how it happened that most amino acids in nature are strictly left-handed, causing the alpha-helix in the proteins they make to be right-handed. It is not accidental that such questions have occupied the minds of biologists and solid-state physicists alike^1^.

In liquid crystals (LC), long-range order can generally be achieved more easily and quickly than in crystals, making them interesting for functional materials where order is required for their function. Such long-range order is normally in molecular orientation and/or position of phase-separated domains (e.g., aromatic and aliphatic). However, in exceptional cases order of the third type, chirality, is also self-propagating in LCs, without the molecules themselves being chiral. This can happen e.g., in layered phases of bent (banana-shaped) molecules. While a stack of upright bananas on a shelf has mirror planes, a stack of tilted bananas has none, hence is chiral, and the domino tilting effect can ensure long-range chiral order^2^.

It has also been discovered recently that two common types of bicontinuous 3D network LC phases are always chiral^3^. These are the triple-network cubic^4,5^ and the tetragonal “Smectic-Q” phase^6^. They form in “polycatenar” rod-like molecules bearing more than one flexible end chain. Their phases consist of column-like segments with the molecules arranged in rafts of two or three lying normal to the column axis. Such segments are joined at three- or four-way junctions. To avoid clashing of the end chains, the successive dumbbell-shaped rafts twist resulting in the segments being helical. The question is why the same twist sense is maintained throughout the entire infinite network. It was proposed^3,5^ that this long-range chiral order is enforced at junctions, where all three or four merging segments must be homochiral, i.e., have the same twist sense, to achieve close packing.

While the bicontinuous network phases are relatively rare^7^, columnar LC phases, with infinite parallel columns and no junctions, are common. Most often they have 2D-hexagonal symmetry (Col_hex_) and form in compounds with disc- and wedge-shaped molecules^8–14^, in honeycomb-forming rod-like amphiphiles with side-chains^15–17^, and in the polycatenars^18–20^. Columnar phases of many compounds are 1D semiconductors and have desirable electro-optic properties when aligned. They can be used e.g., in light harvesting and light emission, in sensors, ionic conductors, etc.^21^. Combining these features with chirality could enhance their versatility as functional materials, e.g., as emitters of circularly polarized light or as membrane materials for separation of left- and right-handed isomers (enantiomers). As columnar mesogens normally have multiple pendant chains, it is likely that the adjacent molecular rafts, or strata, are mutually rotated. However, it is an open question whether uniform twist sense can propagate over long range in junction-free columnar phases as it does in bicontinuous networks. What would eliminate helix reversals and ensure that the same twist sense is maintained?

There have been a number of claims of helical columnar LCs of achiral compounds^22^, some of them ingeniously designed with high barrier to conformational flip (e.g., “propeller-blade” bipyridines^23^. The claims are often based on observed lines perpendicular to the fiber in fiber X-ray patterns (layer lines), but their long-range isochirality has not been proven convincingly. Layer lines, although a feature of diffraction on helices, are just as likely in fiber patterns of completely randomly twisted chains or columns^24^. In the extreme, placing a brick rotated on top of another creates a portion of a helix, but no long-range chirality can be achieved as a random reversal of rotation direction cannot be prevented. Even when the molecules have a chiral group or a chiral dopant is added, while there is amplified preponderance of one helical hand, the length of homochiral sequences is limited and dopant-dependent. This short-range order is characteristic of a paramagnet, with the dopant acting as the external field. However, the question is whether the twisted sense can propagate to true long range on its own in columns of true LCs, including those of achiral compounds. Can column helicity be of ferromagnetic type? Can we have examples of ferrochirality^25^ or even antiferrochirality?

Helix reversals cannot be prevented in an isolated 1D column. In contrast, in column-containing crystals helical sense can propagate as reversals are inhibited by interactions with “correctly” positioned molecules on surrounding lattice; prime examples are various soft-crystal forms of dendronized perylene bisimides (PBIs) bearing wedge-shaped end groups^26^, different “ordered” discotic phases^27^ or in numerous achiral crystalline synthetic polymers^28^. Many helical soft crystals transform to Col_hex_ at higher temperatures, e.g., PBIs and some polymers such as Teflon. Teflon’s chains lose their regular helicity on the transition from crystal to columnar LC at 30 °C^29,30^, probably with soliton-like helix reversals^31^.

Here we report a 3D LC phase where we show that homochirality of helical columns can indeed propagate to long-range even without molecular chirality or network junctions. The phase consists of counter-rotating twisted columns with elliptical or star-like cross-sections. There is long-range order between helical columns but not between molecules, hence it is a liquid crystal and not a crystal. Two of the compounds involved contain a chromophore; the helical configuration enables their efficient π–π stacking, making them interesting for optoelectronic applications. A simple theory based on interacting quadrupoles is developed confirming that, compared to the alternatives, the observed structure is energetically the optimal packing of helices of linear polycatenar dumbbells.

## Results and discussion

### The materials

**Fig. 1:**
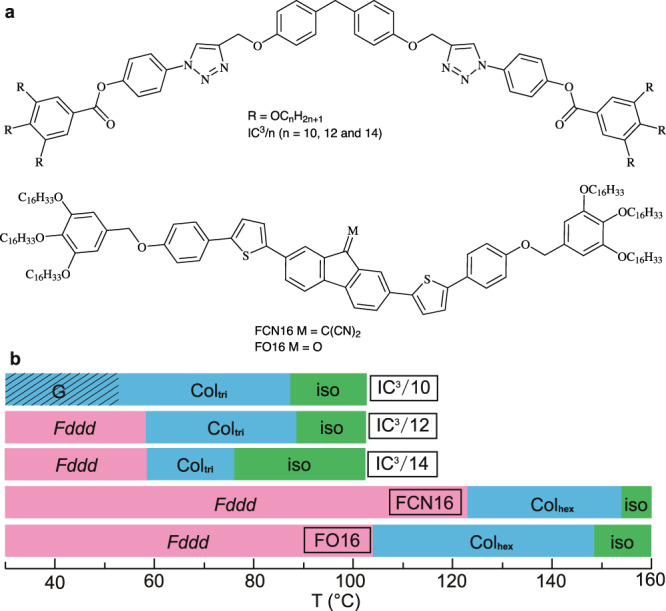
Chemical formulae and phase transition temperatures of the compounds. **a** Chemical formulae of IC^3^/n, FCN16, and FO16. **b** Bar chart of phase transition temperatures of IC^3^/n, FCN16, and FO16.

The *Fddd* phase is found in two types of polycatenars with three chains at each end, one with a straight (FCN16 and FO16) and the other with a bent-core (IC^3^/n—see Fig. 1). The high-T trigonal columnar phase of IC^3^/n compounds was described in ref. ^32^. For synthesis, see “Methods” and Supplementary Methods 1.

**Fig. 2:**
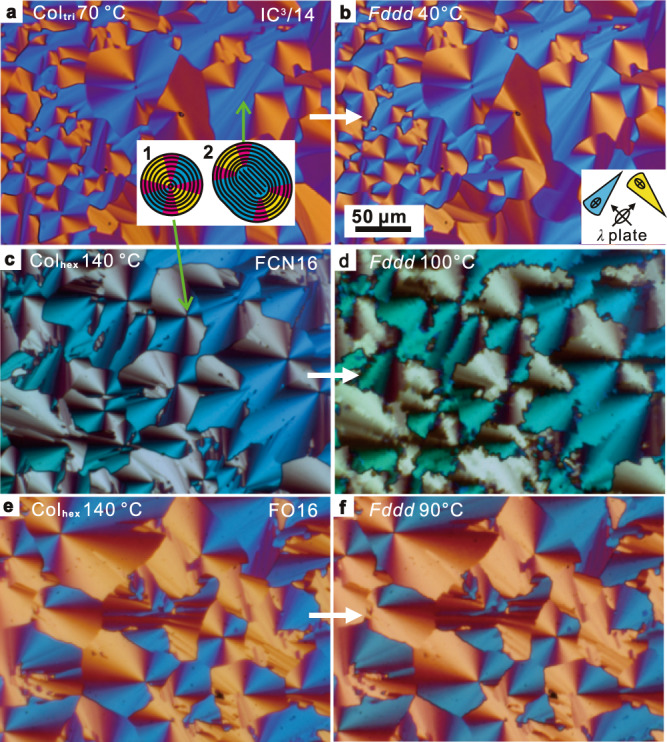
Polarized optical micrographs recorded with a full-wave (*λ*) plate. **a**, **c**, **e** columnar and **b**, **d**, **f** *Fddd* phases of compounds (**a**, **b**) IC^3^/14, (**c**, **d**) FCN16 and (**e**, **f**) FO16. Inset in **a** depicts two developable domains with (1) a *s* = +1 disclination and (2) two +½ disclinations, the latter indicating stiffer columns; black lines are column trajectories. Inset in **b** shows orientation of indicatrices of the *λ*-plate and the colored fans. Scale bar in **b** applies to all Figures. See more textures in Supplementary Section 2.

All compounds form a uniaxial columnar phase at higher temperatures, IC^3^/n with trigonal (Col_tri_)^32^, and FCN16 and FO16 with hexagonal symmetry (Col_hex_), see Table 1, Fig. 1, Figures further below and Supplementary Figs. 1, 7, 8, 10, 11, and 16. Both phases are also referred to collectively as Col. Below Col all except IC^3^/10 also form a 3D LC phase, which is the focus of this study. Its structure is determined by a detailed X-ray study and by atomic force microscopy (AFM), see the next section. Fan (“spherulitic”) textures, typical of columnar developable domains^33^, appear in polarized optical micrographs (POM, Fig. 2 and Supplementary Figs. 2–4). Comparing insets in Fig. 2a, b, we see that the slow axis, hence the aromatic cores, is roughly perpendicular to the column axis. The increase in birefringence following the phase transition is most pronounced in FCN16 (Fig. 2c, d) where, from Michel-Levy color chart, optical retardation increases by 1/3 (from ~150 to ~200 nm), indicating molecular alignment closer to column normal in the low-T phase.

**Table 1:** Phase transition temperatures on cooling.

Name	Phase	Lattice parameters (Å)^a^	*μ*^b^	T/°C [∆*H*/J g^−1^]^c^
IC^3^/10	Col_tri_	*a*_tri_ = 48.9	3	Iso 87 [0.7] Col_tri_ 53 G
IC^3^/12	Col_tri_	*a*_tri_ = 51.3	3	Iso 89 [0.4] Col_tri_ 589 [6.2] *Fddd*
	*Fddd*	*a*_orth_ = 173.5	3	
		*b*_orth_ = 106.1		
		*c*_orth_ = 40.8		
IC^3^/14	Col_tri_	*a*_tri_ = 52.8	3	Iso 769 [0.1] Col_tri_ 59 [7.2] *Fddd*
	*Fddd*	*a*_orth_ = 186.8	3	
		*b*_orth_ = 107.8		
		*c*_orth_ = 40.8		
FCN16	Col_tri_	*a*_hex_ = 54.1	3	Iso 154 [0.8] Col_hex_ 123 [17.0] *Fddd* 28 [46.9] Cr
	*Fddd*	*a*_orth_ = 186.9	2.2	
		*b*_orth_ = 107.9		
		c_o*rth*_ = *34.9*		
FO16	Col_tri_	*a*_hex_ = 53.9	3	Iso 148 [0.3] Col_hex_ 104 [3.9] *Fddd* 29 [9.7] Cr
	*Fddd*	*a*_orth_ = 186.0	2.2	
		*b*_orth_ = 107.4		
		*c*_*orth*_ = *32.5*		

^a^Lattice parameters of hexagonal and trigonal columnar phase *a*_hex_ and *a*_tri_, and orthorhombic *Fddd* phase *a*_orth_, *b*_orth_, and *c*_orth_ (see Supplementary Tables 1, 3–7).

^b^*μ* = number of molecules per column stratum in columnar and *Fddd* phases (see Supplementary Tables 8–9 and Supplementary Discussion 2).

^c^Peak DSC transition temperatures [enthalpies] at 5 K min^−1^ for IC^3^/n and 10 K min^−1^ for FCN16 and FO16. Cr = crystal, G = Glassy, Col_tri_ /Col_hex_ = trigonal/hexagonal columnar, Iso = isotropic melt. For more DSC data, see Supplementary Fig. 1.

### Structure of the mesophase

**Fig. 3:**
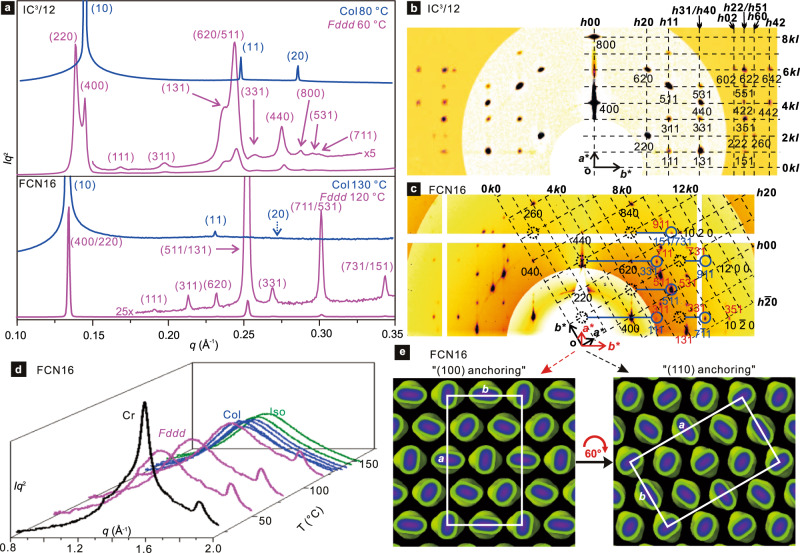
X-ray diffraction results. **a** Transmission powder SAXS curves of Col and *Fddd* phases of IC^3^/12 and FCN16. The dominance of (220) and (400) diffraction peaks show that in both compounds the columns pack on a nearly hexagonal lattice. **b**, **c** GISAXS patterns of *Fddd* phase of IC^3^/12 and FCN16. The background was subtracted and the higher*-q* zone is intensity-enhanced. The partial reciprocal *hk*0 lattice plane is superimposed, with some *hkl* spots in **c** circled blue and connected to their *hk*0 base by blue row lines (for calculation see Supplementary Fig. 9). Reflections in **c** come from two orientations with (110) and (100) planes anchored on Si substrate; reflections in red are from the latter. **d** Evolution of powder WAXS of FCN16 on heating; note the 3.5 Å peak (*q* = 1.8 Å^−1^) in the *Fddd* and crystal phases. **e** Real space FCN16: in both orientations the LC faces substrate with a dense plane of columns (unit cell in white).

**Fig. 4:**
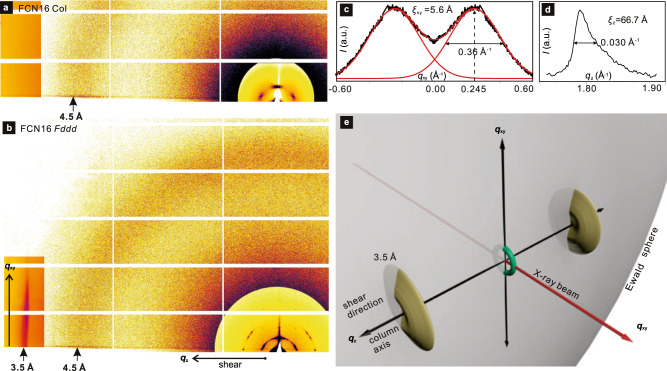
Wider-angle grazing X-ray scattering. **a**, **b** GIWAXS of lightly sheared film of FCN16 in Col (130 °C) and *Fddd* (100 °C) phases, respectively; note the absence of the 3.5 Å streak in Col phase. **c**, **d** *q*_*xy*_ (vertical) and *q*_*z*_ (horizontal) scans of the 3.5 Å streak in **b**; in **c** the peak is reflected across the horizon to facilitate curve resolution. **e** Reciprocal space description of diffraction geometry in **b**, showing symbolically a SAXS ring and the 3.5 Å disks cut by the Ewald diffraction sphere.

The absence of second harmonic (SHG) in the high-T phase of FCN16/FO16 means that the hexagonal Laue symmetry of the small-angle X-ray (SAXS) pattern (Fig. 3a and Supplementary Figs. 8 and 11) comes from a Col_hex_ phase, unlike in IC^3^/n where the presence of SHG indicates trigonal symmetry^32^. As in the Col phase, only a diffuse wide-angle X-ray scattering (WAXS) maximum at 4.5 Å (*q* = 1.40 Å^−1^) is observed for IC^3^/n in the low-T phase (Supplementary Fig. 5b). This confirms the LC nature of the phase. An additional narrower but still diffuse peak appears at 3.4 Å (1.85 Å^−1^) in the low-T phase of FCN16 and FO16 (Fig. 3d and Supplementary Figs. 7 and 10). Its near-meridional position as a vertical streak close to the horizon in the grazing incidence (GIWAXS) pattern of a sheared film (Fig. 4b–e) identifies it as arising from good π–π stacking along *z* (column) axis of parallel aromatic planes.

The transmission powder SAXS patterns of low-T phase of the compounds are rather complex (Fig. 3a and Supplementary Figs. 6a and 12a). The observed diffraction peaks can be indexed to an orthorhombic lattice (*a*~180 Å, *b*~110 Å and *c*~40 Å, Table 1) with the help of grazing incidence SAXS (GISAXS) on surface-oriented thin films (IC^3^/12, Fig. 3b; IC^3^/14, Supplementary Fig. 6b; FCN16, Fig. 3c, Supplementary Fig. 9; FO16, Supplementary Fig. 12b). The GISAXS patterns confirm the reflection conditions *h* + *k*, *h* + *l*, *k* + *l* even, and *h* + *l, h* + *k* equal to 4*n* for *h*0*l* and *hk*0. This narrows the choice of spacegroup to *F*2*dd* (No. 43) or *Fddd* (No. 70, see Supplementary Table 2). As no 0*kl* reflection was observed, the deciding condition *k* + *l* = 4*n* could not be tested. However, since *Fddd* has an inversion centre and *F*2*dd* has not, we performed a second harmonic generation (SHG) test. As can be seen in Supplementary Fig. 15, while the noncentrosymmetric Col_tri_ phase of IC^3^/*n* generates the second harmonic, isotropic liquid and the orthorhombic mesophase do not. This restricts the choice of spacegroup to *Fddd* alone.

**Fig. 5:**
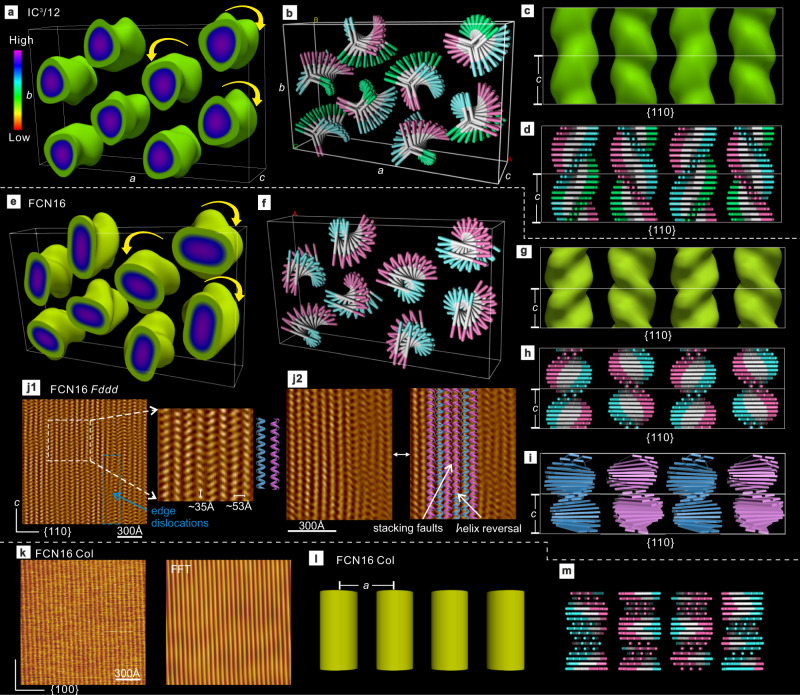
ED maps, schematic models, and AFM images. ED maps and stylized models of *Fddd* phase of (**a**–**d**) IC^3^/12 and (**e**–**h**) FCN16, and (**l**, **m**) of the Col phase of FCN16. **a**, **c**, **e**, **g** 3D maps with high ED regions (aromatic) enclosed within the isoelectronic surface (see also Supplementary Movies 1 and 4). **b**, **d**, **f**, **h** Schematic models of winding rod-like molecular cores (“staircases”, see also Supplementary Movies 2, 3, 5, and 6). The two or three core end groups are colored differently for clarity. The high positional order is grossly exaggerated. For easier comparison of IC^3^/12 and FCN16 structures, the origin of the IC^3^/12 unit cell has been shifted along *b* axis by *b*/4; while IC^3^/12 columns are sitting on 2/1 helical axes, those of FCN16 are on the twofold rotation axes (see Supplementary Fig. 14). **c**, **d**, **g**, **h** View along *b* axis. **i** A more realistic model of **h** taking account of the 8° molecular tilt. **j** AFM of *Fddd* phase of FCN16 recorded at 50 °C (Fourier-filtered height image). The enlarged area shows alternating left- and right-handed columns; stacking faults of mismatched chirality and a helix reversal are indicated in **j2**. **k** original AFM image and inverse FFT of Col phase of FCN16. **l** ED map (time/space average by SAXS) and **m**, random twist model (an instantaneous arrangement) of Col phase of FCN16.

**Fig. 6:**
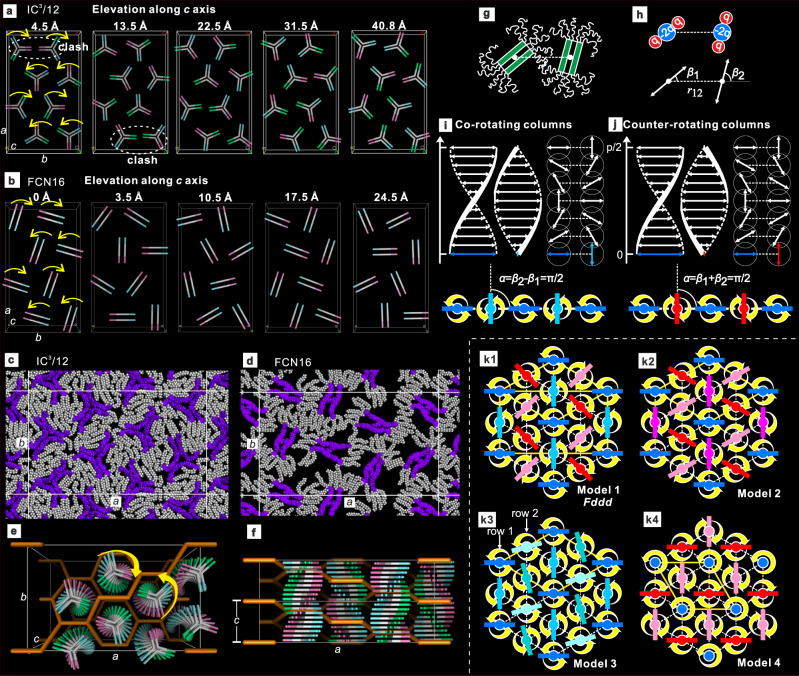
Models of *Fddd* phase and its alternatives. **a**, **b** Layers at different *z*-elevation for (**a**) IC^3^/n and (**b**) FCN16/FO16. **c**, **d** Snapshots of MD-annealed atomistic models (*xy* layer; purple: aromatic, gray/white: aliphatic). **e**, **f** Comparison of the network model of *Fddd* phase in poly(styrene-*b*-isoprene) (yellow rods) with the model of IC^3^/n. Arrows indicate a helical sense of both columns and network. **g**, **h** Two dimers of FO16/FCN16 on neighboring columns in **g** represented by quadrupoles in **h**, where geometrical parameters are also defined. **i**, **j** Side and top views of (**i**) co-rotating and (**j**) counter-rotating ribbons. **k** Minimum-energy configurations on a 2D-hexagonal lattice; **k1** two left**-** and two right-handed columns in a 2 × 2 supercell, equivalent to the *Fddd* structure here observed experimentally; **k2** three left- and one right-handed column in a 2 × 2 supercell; **k3** four right-handed columns in a 2 × 2 supercell; it turns out that vertical shifting of the second row of columns relative to the first does not change the system energy; (**k4**) two left- and one right-handed column in a $\sqrt{3}\times \sqrt{3}$ supercell. The orientation of right-handed columns can be random, which does not affect the system energy (*cf*. structure in ref. ^27^). In **k1**–**k4** right- (left-)handed columns are shades of blue (red).

Electron density (ED) maps were reconstructed from SAXS intensities (Supplementary Section 5 and Supplementary Tables 1 and 5). According to the map of the low-T phase of IC^3^/12 (Fig. 5a, c and Supplementary Movie 1) there are eight columns in the unit cell. As in the Col phase^32^, the calculated number, *μ*, of molecules in each column stratum of thickness 4.6 ± 0.1 Å is 3 (Table 1, Supplementary Table 8, and Supplementary Discussion 2). The cross-section of an averaged column is rounded triangular, similar to that in the Col phase, three bent-core molecules back-to-back forming a three-arm star^32^. However, in contrast to the high-T phase, the orientation of the stars changes monotonically with increasing *z*-elevation, resulting in four left- and four right-handed helical “staircases”. Assuming uniform twist, each consecutive three-arm stratum, or step, is rotated by 13.2° around the column axis—see the model in Figs. 5b, d and 6a. A molecular model of a layer with periodic boundary conditions, using the experimental lattice constants and subjected to 30 cycles of molecular dynamics (MD) annealing is shown in Fig. 6c; note efficient space-filling.

The ED map of the *Fddd* phase of FCN16 (Fig. 5e, g and Supplementary Movie 4) shows even more clearly the twist of the columns, here appearing like twisted ribbons. The column section is now oval, with close to *μ* = 2 parallel linear molecules per column stratum. This is referred to below as “dimer” (Table 1 and Supplementary Table 8); see also Fig. 5f and the MD snapshot in Fig. 6d. Even though the strata are thinner than in IC^3^/n (3.4–3.5 Å), the twist angle between strata in the linear compounds is larger (18–19°) than that in IC^3^/*n* (13°) (see Supplementary Table 10), making the pitch (=2*c*) considerably shorter—compare models in Fig. 5d, h.

The discontinuous drop in *μ* in a thermal transition, as observed here from 3 to ∼2 in FO16 and FCN16, is rare but not unique in columnar phases. A discontinuous drop from *μ* = 4 to 3.5 has been observed in pizza-slice-shaped molecules of trialkoxybenzoate salts (“dendron ejection transition”)^34^, a process that can even result in unusual columnar superlattices^35^.

All columns in (100) rows along *b* axis co-rotate, either clockwise or anticlockwise, but along {110} diagonals they counter-rotate, alternating between left and right helicity (Fig. 5a–h). *xy* sections of the model at different *z*-elevations (Fig. 6a, b and Supplementary Figs. 17 and 18) show that the linear dimers of FO16 and FCN16 succeed in evading clashes at every level (see Supplementary Movie 6), while the co-rotating stars of IC^3^/n unavoidably clash along *b* at *z* = 4.5 Å and *z* = 13.5 Å (see Supplementary Movie 3). Interestingly, while the *a/b* ratio for FCN16, FO16, and IC^3^/14 is exactly √3 (186.9/107.9, 186.0/107.4, 186.8/107.8), meaning that the column axes sit precisely on a hexagonal lattice, for IC^3^/12 the ratio is 1.635, i.e., the unit cell is stretched along the clash direction *b* (Fig. 6a). The deviation from hexagonal grid also causes separation of (400) and (220) reflections (Fig. 3a). Probably the “padding” by the C12-chains is insufficient to fully mitigate the clashes. Significantly, IC^3^/10 does not form *Fddd* at any temperature (Table 1).

The alternating helices in *Fddd* phase are also observed directly by AFM on FCN16 (Fig. 5j and Supplementary Figs. 20 and 21). Columns without helicity are observed in the Col phase in Fig. 5k, matching the ED map and model in Fig. 5l, m. The ~53 Å distance between helical columns in Fig. 5j and the ~35 Å helical pitch match almost exactly the ED map and the proposed structural model of *Fddd*. Edge dislocations, stacking faults of mismatched chiral columns, and helix reversal defects are also seen in Fig. 5j.

An interesting consequence of the different column cross-sections in IC^3^/n and FCN16/FO16 appears to be a higher bending modulus of the star-like IC^3^/n columns compared with that of the bendable ribbons of FCN16/FO16. The difference is evident in the type of disclinations in POM images in Fig. 2; only FCN16/FO16 columns can tolerate the high curvature at the center of a *s* = +1 disclination (Fig. 2a, inset 1). In contrast, the stiffer IC^3^/n columns prefer ends to sharp bends. Thus the central column circle splits into two semicircles with a larger radius (two *s* = +1/2 disclinations), with a bundle of straight but finite length columns filling the central part (Fig. 2a, inset 2).

### 3D order of helices, not molecules

The high surface alignment in GISAXS/GIWAXS experiments allows some further structure refinement and clarification of the true nature of the mesophase. Significantly, the 3.4 Å peak in powder WAXS (Fig. 3d) corresponds to the streak at *q*_*z*_ = 2π/3.5 Å in GIWAXS of columns aligned parallel to substrate (Fig. 4b, Supplementary Fig. 13b). The 0.1 Å difference comes from the 8° tilt of the aromatic planes from the *xy* plane, causing a displacement of the intensity maximum away from the *z*-axis (horizon); see *I*(*q*_*xy*_) intensity profile, Fig. 4c. The tilt is taken into account in the model in Fig. 5i, which is more realistic than the simplified version in 5 h.

Notably, while there are many small-angle Bragg reflections with resolution-limited width, i.e., with correlation lengths of at least 1800 Å (domain size >5000 Å), there are no Bragg reflections at wide angles. This means that *Fddd* is a true LC and not a crystal. A more precise distinction is provided by the 3.5 Å streak. From the *q*_*z*_-profile the correlation length along the columns is ${{\xi }}_{z}=2/\triangle {q}_{z}$ = 67 Å (Fig. 4d). However, from the *q*_*xy*_-profile along the streak, we get ${{\xi }}_{{xy}}=2/\triangle {q}_{{xy}}$=5.6 Å, or “crystal size” of π*ξ*_*xy*_ = 18 Å, which is smaller even than the width of one single column (>50 Å). These measurements confirm that the *Fddd* is a true LC and not a crystal, soft or otherwise, as in many reported helical column-like soft structures. Thus, while there is true 3D long-range order in packing of helical columns, there is no intercolumnar correlation between positions of individual molecules. One may think of the *Fddd* phase as consisting of orderly interlocked helical tubes filled with mobile molecules with no preferred position and no lattice. A somewhat similar situation has been observed previously in a chiral helicene, but with substantially lower order in “tube” packing^36^.

**Fig. 7:**
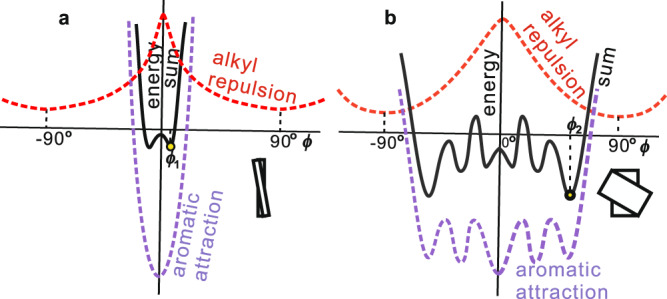
Schematic depiction of the interaction energy between successive molecules at adjacent *z*-elevations (strata) within an isolated column as a function of angle of rotation *ϕ* about the column axis. **a** For molecular rods one benzene ring wide (present case), **b** for disc-like or elongated aromatic platelet-like mesogens containing more than one ring in both *x* and *y* directions. The solid black line *S*(*ϕ*) is the sum of energies of attraction between the π-conjugated molecular cores *A*(*ϕ*) and the repulsion between alkyl end chains *R*(*ϕ*). For some actual examples of *A*(*ϕ*) see ref. ^37^. Yellow circles indicate global energy minima causing a small twist angle *ϕ*_1_ in rod-like and a larger twist angle *ϕ*_2_ in disc/platelet-like mesogens.

According to our model, there is a fundamental difference between helical columns assembled from rod-like molecules on the one hand and those from disc- and plate-like molecules on the other. For rod-like molecules such as those studied here, which include both straight and bent rods, the twist angle *ϕ* between successive rafts, or strata, in a column is a balance between the steric repulsion *R*(*ϕ*) between the alkyl brushes at rod ends (red dashed curve in Fig. 7a) and the attraction *A*(*ϕ*) between the π-conjugated rods (blue dashed curve). *R*(*ϕ*) has a maximum when the rods are parallel (maximum clash, *ϕ* = 0) and a minimum when the rods are perpendicular (*ϕ* = 90°). *A*(*ϕ*) has a sharp minimum when the rods are parallel (*ϕ* = 0°) as there is maximum π-π overlap along the entire length of the rods. The resulting sum energy function *S*(*ϕ*) (black solid line) therefore has two minima at ±*ϕ*_1_ where *ϕ*_1_ is a small angle. In the *Fddd* phase the interaction with neighboring columns will break the degeneracy, making the two minima unequal, thus favoring one twist sense over the other—see the separate section on column packing below. Due to the molecular motion in the LC, the averaged columns are smooth twisted ribbons.

In contrast, the situation is different where discs or plate-like mesogens two or more benzene rings wide are involved. Here *A*(*ϕ*) has minima additional to that at *ϕ* = 0, because by rotating the successive platelets around the column axis further aromatic rings will come in contact producing local minima (Fig. 7b). The twist angle *ϕ*_2_ will then be fixed by the global minimum in the wavy sum function *S*(*ϕ*) = *R*(*ϕ*) + *A*(*ϕ*). *ϕ*_2_ will typically be considerably larger than *ϕ*_1_ in narrow rod molecules, as seen in disc-like^37^ or elongated plate-like mesogens such as PBI^26,38^, or in hat-like molecules like cyclotriveratrylene^39^. Instead of being a continuous helix, such knobbly columns are discontinuous helices that tend to interlock with neighboring columns resulting in crystals with 3D long-range positional order of molecules.

### Antiferrochirality

Spontaneous long-range alignment modes of electric dipoles in SmC* LCs (layers of tilted chiral molecules) and in some bent-rod LCs, are well known, respectively, as ferroelectric or antiferroelectric^40^. In the same vein, it is useful to regard the present *Fddd* phase as “antiferrochiral”. This helps distinguishing it from the reported cases of helical columns, where the range of homochirality is short and dependent on the possible presence of chiral substituents or dopants, which act as external field acts on a paramagnet. The well-known frustration of antiferromagnets on a triangular lattice is also present here, causing symmetry-breaking of hexagonal to orthorhombic.

### Comparison with cubic LCs and *Fddd* phase in block copolymers

A phase with *Fddd* symmetry, albeit with a distinctly different structure, has been reported previously in block copolymers (BCPs)^41,42^. The *Fddd* lattice parameters (*a* is defined as the largest lattice parameter as used in this paper, and in the following parameters from literature have been changed accordingly for easier comparison.) in poly(styrene-*b*-isoprene) or poly(isoprene-*b*-styrene-*b*-ethyleneoxide) are naturally an order of magnitude larger, *a* varying from 750 to 2125 Å^43^. However, the *a*:*b* ratio is similar to that in our LCs, about $\sqrt{3}\!:$1 (Supplementary Table 11). Unlike our LC phase which consists of columns, the *Fddd* in BCPs is a single network bicontinuous phase with 3-way junctions, as shown in orange, scaled, in Fig. 6e, f and Supplementary Fig. 19, together with our IC^3^/12 model. In *Fddd* phase of PS-*b*-PI, PS forms the network embedded in PI^34^. In a crude analogy, the aromatic cores in our model can be said to occupy PI-rich areas in the BCP phase. Evidently, the IC^3^/n helical columns fill the space between network segments, with the twist sense of the columns matching that of the surrounding network. The *Fddd* phase in BCPs is found between lamellar and Cub_bi_ phases (${Ia}\bar{3}d$ or *I*4_1_32)^42^, while our *Fddd* appears below the columnar.

The chiral columns in *Fddd* phase remind us of helical segments of networks forming the bicontinuous phases mentioned in the introduction. These are the double-gyroid cubic ${Ia}\bar{3}d$ phase^7^, the triple-network cubic *I*23^4^ or the tetragonal “Smectic-Q” (*I*4_1_22)^6^. While the latter two are chiral even in achiral compounds, the two networks in the ${Ia}\bar{3}d$ have opposite hands, resulting in no overall chirality. The gyroid can thus be considered antiferrochiral and the triple-network and the SmQ ferrochiral. In all three phases the twist between successive molecular strata is 8–10°. As in the *Fddd*, this twist balances the attraction between the cores *A*(*ϕ*) and the repulsion *R*(*ϕ*) of the end chains. Since the compounds forming the above bicontinuous phases contain only 3–4 end chains, their smaller twist than the ~18° in the six-chain FCN16/FO16 is understandable.

In bicontinuous phases, the homochirality of each network is enforced at junctions, where all three or four merging helical segments must be homochiral to minimize steric clash. However in the *Fddd* there are no junctions and the columns are parallel. What propagates their homochirality is the 3D order of interlocking helices which ensures that helix reversals, such as that in Fig. 5j2, are eventually corrected by the surrounding lattice. In columnar phases of covalent or self-assembled discs columns have cylindrical symmetry, hence such 3D interlock is unlikely. The present results thus suggest that a noncircular column cross-section, such as oval or star-like, is beneficial in facilitating 3D interlock that could sustain homochirality.

### A simple theory of packing of helices: *Fddd* and alternative models

The interaction between two FCN16/FO16 dumbbell dimer rafts from neighboring columns at the same elevation can be described quantitatively as that between two linear quadrupoles oriented at *β*_1_ and *β*_2_ (Fig. 6g, h). The repulsion of equal charges at the ends of the rod-like quadrupole represents the steric repulsion of the pendant flexible chains. Considering each helix as a series of such twisting quadrupoles, the interaction energy *E* between two neighboring columns is the sum of interactions between their corresponding quadrupoles at all elevations. Our calculations (Supplementary Discussion 1) show that *E* is determined by constant α, defined as $\alpha ={\beta }_{2}-{\beta }_{1}$ for co-rotating and $\alpha ={\beta }_{1}+{\beta }_{2}$ for counter-rotating columns (Fig. 6i, j).

For a row of columns, the minimum *E*, *E*_*min*_, is found for counter-rotating columns with $\alpha ={\beta }_{1}+{\beta }_{2}=\pi /2$ (Fig. 6j). If all columns must co-rotate, a higher *E*_*min*_ has $\alpha ={\beta }_{2}-{\beta }_{1}=\pi /2$ (Fig. 6i). In $\frac{3{\phi }^{2}}{32{r}_{12}^{5}}$ units, where *ϕ* is the quadrupole moment and *r*_12_ the distance between quadrupoles*, E*_*min*_ per dimer/quadrupole is −29 in a counter-rotating, 3 in a co-rotating, and 6 in a random row. However, the condition to keep all neighboring columns counter-rotating cannot be satisfied on a 2D-hexagonal lattice. The obvious choice then is to keep as many counter-rotating neighbors as possible, as shown in Fig. 6k1. A 2 × 2 supercell is assumed, as both counter- and co-rotating minimum-energy rows have a two-column repeat (Fig. 6i, j). We have two left- and two right-handed helices in the 2 × 2 supercell, and each has four counter-rotating and two co-rotating neighbors. Energy minimization shows that it is impossible to have $\alpha =\pi /2$ for all rows simultaneously; instead the best solution has $\alpha =\pi /2$ for co-rotating and $\alpha =\frac{5\pi }{12}$ for counter-rotating columns (Fig. 6k1), with interaction energy per dimer about −45.6 (Supplementary Table 13). Co-rotating columns having $\alpha =\pi /2$ means that neighboring helices are vertically shifted by quarter-pitch (Fig. 6i). This results in a 3D orthorhombic cell with *Fddd* symmetry, fitting almost exactly our observed dimer orientations at different elevations (*cf*. Fig. 6k1 and b/3.5 Å).

Other possible arrangements of helical columns have also been explored (Fig. 6k2–k4). The energies for models 1–4 were −45.6, −36.75, 15, and 15, respectively. The energy of Model 1 with two LH and two RH rotations, our proposed *Fddd* structure, was clearly the lowest. In comparison, the calculated energy for a completely uncorrelated Col phase is 18. For more details, see Supplementary Discussion 1. It is therefore concluded that the antiferrochiral *Fddd* structure is a result of packing optimization of helical columns with a noncircular cross-section.

It is also interesting to note that the homochiral Model 3, with four ribbons in a unit cell, all twisted in the same direction and which represents a ferrochiral structure, has high energy and is therefore unlikely to form spontaneously in non-chiral compounds. At the same time, however, the superlattice Model 2 with 3 LH and 1 RH column (or vice versa), having an enantiomeric column ratio 3:1, and thus being chiral, has an energy not too much higher than the *Fddd*. There is thus a possibility that it might be observed in some systems in which other structural factors would override the energy disadvantage calculated by our simple model. Being equivalent to a ferrimagnet, its structure could be regarded as ferrichiral. In fact, it mirrors precisely the prototype ferrimagnet Fe_3_O_4_, which has three Fe moments parallel and one antiparallel.

### Spectra and π–π stacking

**Fig. 8:**
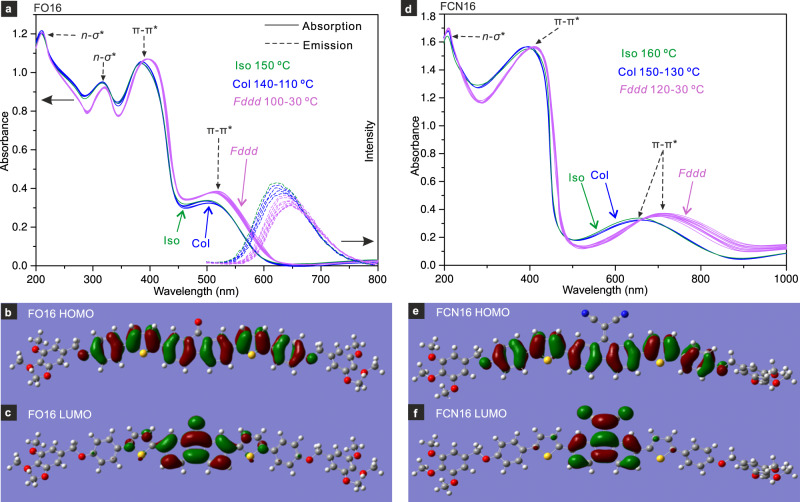
UV–vis–fluorescence spectra and HOMO-LUMO orbitals. **a** UV–vis and fluorescence emission spectra and **b** HOMO and **c** LUMO orbital isosurfaces of FO16. **d** UV–vis, **e** HOMO, and **f** LUMO orbitals of FCN16. Spectra are recorded during cooling from isotropic phase. FO16 emission is excited at 420 nm. HOMO and LUMO energies and bandgaps calculated by DFT (B3LYP/6-31G) are *E*_*HOMO*_ = −5.02 eV, *E*_*LUMO*_ = −2.25 eV, Δ*E* =  2.77 eV for FO16 and *E*_*HOMO*_ = −5.11 eV, *E*_*LUMO*_ = −3.12 eV, Δ*E* = 1.99 eV for FCN16. The bandgaps measured from vis spectra by the Tauc plot method^49^ are 2.15 eV (Col) and 2.10 eV (*Fddd*) for FO16, and 1.52 eV (Col), and 1.35 eV (*Fddd*) for FCN16 (see Supplementary Table 12). FCN16 is expected to emit in near IR (not measured).

With conjugated fluorophore cores, FCN16 and FO16 are potential electroptic materials^44,45^. Their UV–vis and fluorescence spectra are shown in Fig. 8 together with HOMO and LUMO orbitals calculated by density functional theory (DFT). The bandgaps measured from FO16 and FCN16 spectra in Col phase are 2.15 and 1.52 eV, smaller than calculated (Fig. 8 and Supplementary Table 12) due, at least partly, to the neglect of intermolecular conjugation in the calculation. The bandgaps narrowed by a further 0.07 and 0.17 eV on Col-*Fddd* transition, causing red shifts of ∼20 and 50–60 nm in FO16 and FCN16. These results indicate an increase in intermolecular conjugation upon the transition. In the Col phase 4.5 Å thick three-molecule strata orient randomly in the *xy* plane (Figs. 4a and 5l, m and Supplementary Fig. 13a). On Col-*Fddd* transition the number of molecules drops to about two and the inter-strata spacing to 3.5 Å.

As mentioned in Supplementary Discussion 2, the difference between the ∼4.5 Å and ∼3.5 Å spacing in columnar phases is usually associated with the change from the T-type stacking mode (aromatic planes on successive molecules close to perpendicular) to the parallel mode (aromatic ring planes nearly parallel). In FO16 and FCN16, this change upon the Col*-Fddd* transition is also consistent with the change to a more strongly negative birefringence (rings closer to perpendicular to the column; Fig. 2c, d) and a narrowing of the bandgap. These changes are absent in IC^3^/n compounds which maintain the T-type stacking in both phases.

The particularly large bandgap narrowing in FCN16 is consistent with the potent malononitrile acceptor (Hammett constant for CN σ_p_ = 0.66 (http://www.wiredchemist.com/data/hammett-sigma-constants) interacting strongly with the electron-rich adjacent aromatic cores^46^ and stabilizing the π-stack^47^. FCN16 stands out also by its large transition enthalpy (17J/g) and increase in birefringence (Fig. 2c, d). The near-IR fluorescence, expected in FCN16, could be useful in achieving greater light penetration through opaque tissue^48^.

In summary, a complex 3D liquid-crystal phase is discovered in bent- and straight-core compounds, having orthorhombic *Fddd* symmetry and consisting of counter-rotating helical columns. Unlike previous reports of helical columnar LCs, in spite of being non-crystalline, helical periodicity is long-range and spontaneous. As antichiral near-neighbor interactions are favored, the structure is equivalent to an antiferromagnet, hence termed antiferrochiral. Its discovery confirms the universality of the principle of twisting polycatenar columns as building blocks for complex 3D self-assembly, previously established in bicontinuous network LCs, albeit here without network junctions. The long-range twist sense is shown to propagate through mere steric inter-helical interaction. Comparison of different helical packing models based on linear quadrupoles confirms that in achiral compounds the antiferrochiral *Fddd* is the lowest energy solution, while the ferrochiral alternative is energetically the least favored.

Other helical column phases could be designed on similar principles. As the chiral Model 2 structure, with a 3:1 ratio of column enantiomers, has an energy not too high above that of the *Fddd*, by modifying the molecule it may be possible to obtain it by spontaneous assembly. With suitable fluorophores this could lead to new organic circularly polarized LEDs, enantioselective filters or sol-gel templates for chiral porous ceramics.

## Methods

### Synthesis

IC^3^/n compounds were synthesized by a Cu(I)-catalyzed click reaction^32^. Synthesis and chemical characterization of the straight-core compounds FO16 and FCN16 is described in Supplementary Methods 1.

Reactions requiring an inert gas atmosphere were conducted under nitrogen and the glassware was oven-dried (105 °C). Tetrahydrofuran (THF) was distilled from sodium prior to use. Commercially available chemicals were used as received. ^1^H-NMR and ^13^C-NMR spectra were recorded on a Bruker-DRX-300 spectrometer and a Bruker-DRX-400 spectrometer. The mass of the compounds was characterized by matrix-assisted laser desorption/ionization time-of-flight mass spectrometry (MALDI-TOF-MS) on a Bruker autofleX max instrument. Elemental analysis was performed using an Elementar VARIO EL elemental analyzer. Thin-layer chromatography was performed on aluminum plates precoated with 5735 silica gel 60 PF254 (Merck). Column chromatography was carried out on Merck silica gel 60 (230–400 mesh).

Transmission powder SAXS/WAXS experiments were carried out at station I22 of Diamond Light Source, U.K. Powder samples in 1-mm glass capillaries were held in a modified Linkam hot stage. Pilatus 2 M detector (Dectris) was used and the X-ray energy was 12.4 keV. GISAXS/GIWAXS experiments were done at BM28 of European Synchrotron Radiation Facility, France, and I16 of Diamond Light Source. 2D diffraction patterns were collected using a MAR165 CCD camera at BM28 and Pilatus 2 M at I16. Thin-film samples were prepared from melt on the silicon substrate. *n*-tetracontane was used to calibrate the sample to detector distance.

Electron density maps were calculated by inverse Fourier transformation using the standard procedure as described in International Tables for Crystallography. Integral intensities of all peaks were measured using Gaussian peak fitting. GISAXS intensities were used to help resolve overlapping peaks. More details are in Supplementary Information.

Molecular models were built using Materials Studio (Accelrys). Geometry optimization and molecular dynamic annealing were performed using Forcite Plus module with Universal Force Field. NVT annealing dynamics was performed through 30 cycles between 300 and 600 K, with a total annealing time of 30 ps. Frontier orbital distributions of fluorene derivatives were calculated using the DFT at level B3LYP, 6–31 G(d) basis set.

AFM imaging was done in tapping mode on a Bruker Multimode 8 instrument with Nanoscope V controller. The sample was dissolved in toluene and spin-coated on a highly ordered pyrolytic graphite substrate.

Samples of ca. 50-μm thickness for frequency-doubled light emission, or second harmonic generation (SHG) measurements were prepared on the rough side of silicon wafer and examined using a Zeiss LSM 510 Meta upright laser-scanning confocal microscope with a ×40/0.75NA objective. The temperature was controlled by a Linkam hot stage. A Chameleon Ti:Sapphire femtosecond pulsed laser (Coherent, California), tuned to 800 nm, was attached to the microscope and focused onto the sample resulting in a SHG signal detectable at 400 nm. In order to exclude possible surface effects, first the beam was focused at the bottom of the LC film in contact with the Si substrate, then at 20 μm height. The SHG ratio was calculated by dividing the intensities at 400 nm by the averaged background around the peak (see also ref. ^32^).

For UV–vis and fluorescence emission spectroscopy, the samples were melted and spread evenly between quartz plates (1 mm thickness). UV–vis spectra were recorded using Lambda 900 (Perkin Elmer). Fluorescence spectra of FO16 were excited at 420 nm and recorded by Fluoromax4 (Horiba).

DSC thermograms were recorded on a DSC 200 F3 Maia calorimeter (NETZSCH) with heating/cooling rates as specified.

Polarized optical micrographs were recorded using an Olympus BX-50 equipped with a Mettler HS82 hot stage.

## Supplementary information

Supplementary Information

Description of Additional Supplementary Files

Supplementary Movie 1

Supplementary Movie 2

Supplementary Movie 3

Supplementary Movie 4

Supplementary Movie 5

Supplementary Movie 6

## Data Availability

The raw GISAXS/GIWAXS data (tiff files) of this study are available for free download from the Figshare database: 10.6084/m9.figshare.15027246.

## Code availability

All derived mathematical equations are given in Supplementary Information and no special code was required in their use.
