# Supplementary material for: A case of antiferrochirality in a liquid crystal phase of counter-rotating staircases
Source: Nat Commun. 2022 Jan 19;13:384. doi: 10.1038/s41467-022-28024-1 (PMC8770800; doi:10.1038/s41467-022-28024-1)
Supplement: Supplementary file 1 — Supplementary Information [file 41467_2022_28024_MOESM1_ESM.pdf]

Supplementary Information

A case of antiferrochirality in a liquid crystal phase of counter-rotating staircases

Ya-xin Li[1,2,♣], Hong-fei Gao[3], Rui-bin Zhang[2], Kutlwano Gabana[4], Qing Chang[3], Gillian A. Gehring[4], Xiao-hong Cheng[3,5]*, Xiang-bing Zeng[2]* and Goran Ungar[1,2]*

1. State Key Laboratory for Mechanical Behaviour of Materials, Shaanxi International Research Centre for Soft Matter, Xi'an Jiaotong University, Xi'an, 710049, P. R. China. Email: g.ungar@xjtu.edu.cn; g.ungar@sheffield.ac.uk
2. Department of Materials Science and Engineering, University of Sheffield, Sheffield S1 3JD, UK. E-mail: x.zeng@sheffield.ac.uk
3. Key Laboratory of Medicinal Chemistry from Natural Resources, Ministry of Education, Yunnan University, Kunming, P. R. China. E-mail: xhcheng@ynu.edu.cn
4. Department of Physics and Astronomy, University of Sheffield, Sheffield E1 2C, UK.
5. School of Chemistry and Chemical Engineering, Yangtze Normal University, Fuling, P. R. China.

♣ Present address: School of Chemistry and Chemical Engineering, Henan University of Technology, Zhengzhou 450001, P.R. China.

Table of Contents

Supplementary Information **1**

Supplementary Section 1. DSC thermograms 2

Supplementary Section 2. Polarized optical microscopy (POM) 2

Supplementary Section 3. X-ray diffraction data 4

Supplementary Section 4. Second harmonic generation 12

Supplementary Section 5. Reconstruction of electron density maps 13

Supplementary Section 6. Estimate of the number of molecules per column stratum 14

Supplementary Section 7. Additional schematic models with ED maps 16

Supplementary Section 8. Additional AFM images 18

Supplementary Section 9. UV-vis and fluorescence emission spectroscopy 20

Supplementary Discussion 1. Minimum energy packing of helical columns 21

Supplementary Discussion 2: Comments on the number of molecules per column stratum 25

Supplementary Methods. Synthesis and analytical data 27

Supplementary References 33

Supplementary Section 1. DSC thermograms

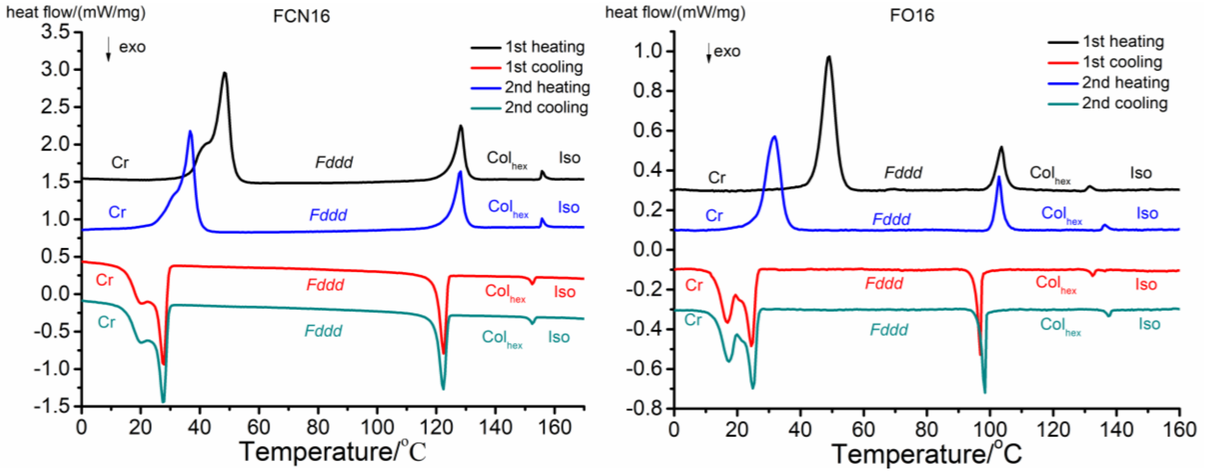

Supplementary Figure 1. DSC heat flow thermograms (exo down) of compounds FCN16 and FO16 at heating and cooling scan rate of 10 K min^{-1}.

Supplementary Section 2. Polarized optical microscopy (POM)

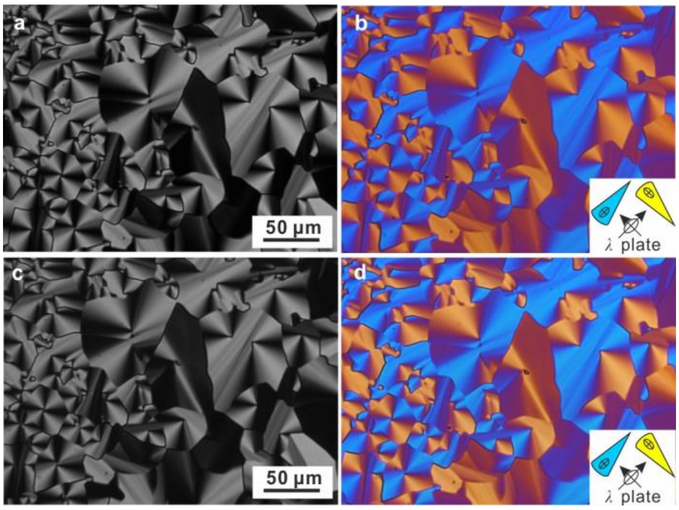

Supplementary Figure 2. POM of $IC^3/14$ examined at **a**, **b** 70 ºC and **c**, **d** 40 ºC, at which temperatures the sample was in Col and *Fddd* phases, respectively. (**b**, **d**) are recorded with a full-wave (*λ*) plate.

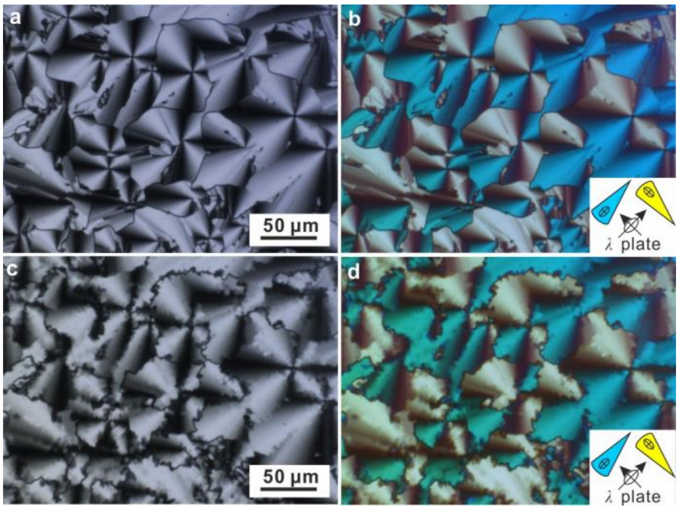

Supplementary Figure 3. POM of FCN16 examined at **a**, **b** 140 ºC and **c**, **d** 100 ºC, at which temperatures the sample was in Col and *Fddd* phases, respectively. (b, d) are recorded with a full-wave (*λ*) plate.

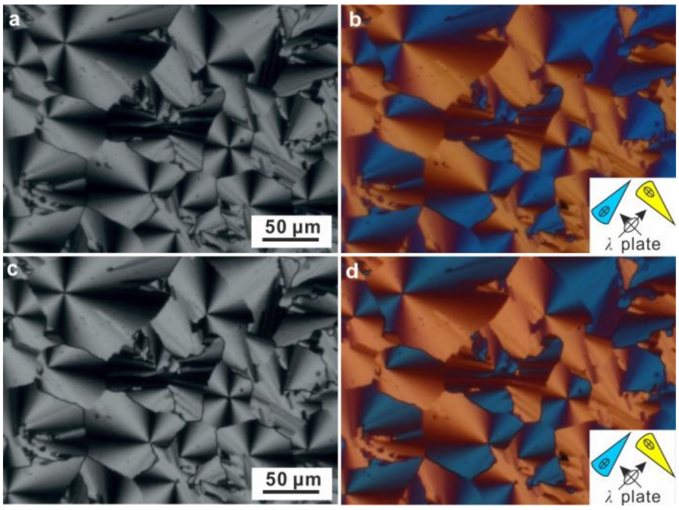

Supplementary Figure 4. POM of FO16 examined at (a,b) 140 ºC and (c, d) 90 ºC, at which temperatures the sample was in Col and *Fddd* phases, respectively. (b, d) are recorded with a full-wave (*λ*) plate.

Supplementary Section 3. X-ray diffraction data

$IC^3/12$

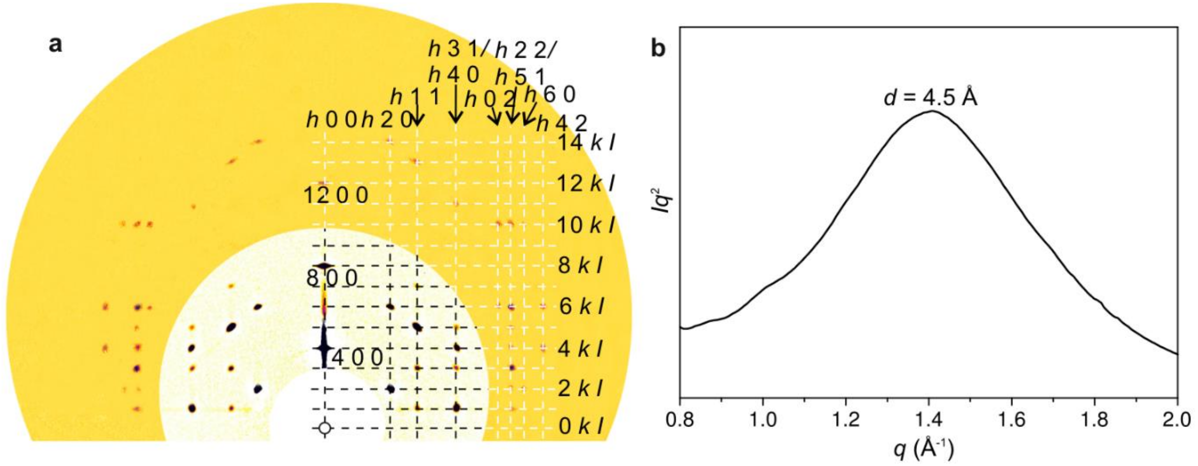

Supplementary Figure 5. a, GISAXS pattern and **b**, transmission powder WAXS curve of *Fddd* phase of $IC^3/12$ recorded at 60 ºC. Fitted background has been subtracted separately from the inner (white) and outer (yellow) portions of the GISAXS pattern.

Supplementary Table 1. Indices, experimental, calculated *d*-spacings, intensities, phases and lattice parameters of diffraction peaks of $IC^3/12$ recorded by transmission powder SAXS in the *Fddd* phase at 60 °C. All intensities are Lorentz and multiplicity corrected.

(*hkl*)	experimental *d*-spacings (Å)	calculated *d*-spacings (Å)	*Intensity*	*Phase*
(220)	45.2	45.3	93.0	0
(400)	43.3	43.4	100.0	π
(111)	37.2	37.2	0.4	-
(311)	31.7	31.8	0.4	-
(131)	26.4	26.4	4.0	π
(511)[a]	25.6	25.6	6.4	0
(620)			3.8	π
(331)	24.3	24.3	0.2	-
(440)	26.8	26.3	3.9	π
(800)	21.7	21.7	1.2	-
(531)	21.2	21.2	0.1	-
(711)	20.8	20.8	0.1	-
a = 173.5 Å, b = 106.1 Å and c = 40.8 Å				

a: (511) and (620) diffraction peaks are overlapping in the powder diffraction pattern. While their total intensity is taken from the powder diffraction curve, the intensity ratio between the two has been determined from the GISAXS pattern. An azimuthal scan through the (511) and (620) diffraction peaks in the 2D GISAXS pattern is obtained first, and the intensities of the two diffraction peaks are measured from the areas of the diffraction peaks in the scan by fitting each peak to a Gaussian function. Due to the sample geometry, equivalent to a fibre pattern (multiple domains sharing the same *a*-axis), measured intensities needed to be multiplied by the corresponding q_{yz}, i.e. q vector component which is perpendicular to the common *a*-axis of the domains. For (511) and (620) these are $q_{(011)}$ and $q_{(020)}$ respectively. Further correction by diffraction peak multiplicity leads to an intensity ratio $I_{(511)} : I_{(620)}$ of 63:37.

Supplementary Table 2. Reflection conditions and space groups.

Reflection conditions							Space group
hkl	*h*0*l*	*hk*0	0*kl*	*h*00	0*k*0	00*l*	
h + *k*, *h* + *l*, *k* + *l*	*h* + *l*	*h* + *k*	*k* + *l*	*h*	*k*	*l*	*F*222, *Fmm*2, *Fm*2*m*, *F*2*mm*, *Fmmm*,
	h, *l*	*h* + *k* = 4n; *h*, *k*	*k* + *l* = 4n; *k*, *l*	*h* = 4n	*k* = 4n	*l* = 4n	*Fd*2*d*
	h + *l* = 4n; *h*, *l*	*h*, *k*	*k* + *l* = 4n; *k*, *l*	*h* = 4n	*k* = 4n	*l* = 4n	*Fdd*2
	h + *l* = 4n; *h*, *l*	*h* + *k* = 4n; *h*, *k*	*k*, *l*	*h* = 4n	*k* = 4n	*l* = 4n	*F*2*dd*
	h + *l* = 4n; *h*, *l*	*h* + *k* = 4n; *h*, *k*	*k* + *l* = 4n; *k*, *l*	*h* = 4n	*k* = 4n	*l* = 4n	*Fddd*

* *h* + *k* means the value of *h* + *k* is even; *h*, *k* and *l* means the values are even.

$IC^3/14$

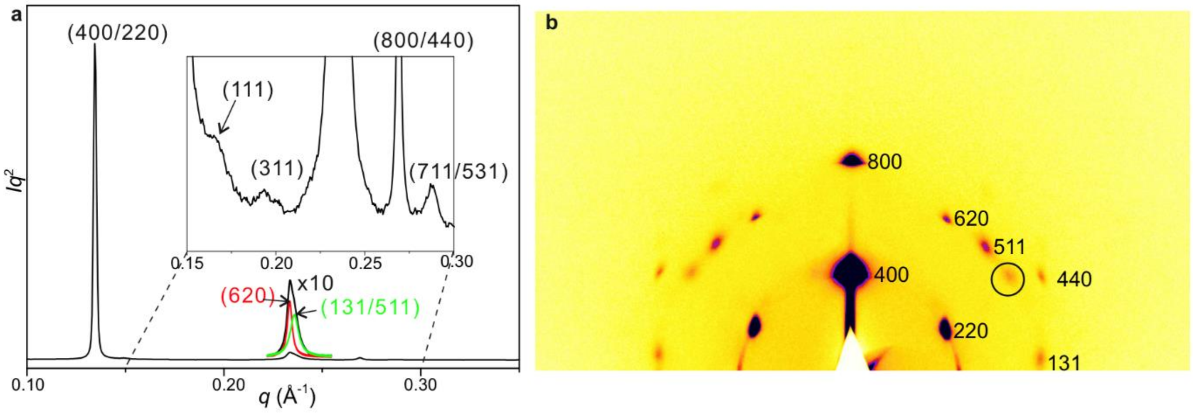

Supplementary Figure 6. a, Powder SAXS curve and **b**, GISAXS pattern of *Fddd* phase of $IC^3/14$ recorded at 60 °C (cooled from 80 °C). The black circled spots are (511) from the other orientation. More details of the other orientation can be seen in Supplementary Figure 10. As the *d*-spacings of (620) and (511/131) are close with each other, it is shown in one peak in powder SAXS. The separated and fitted peaks are displayed in the figure.

Supplementary Table 3. Indices, experimental, calculated *d*-spacings, intensities, phases and lattice parameters of diffraction peaks of IC[3]/14 recorded by transmission powder SAXS in the *Fddd* phase at 60 °C (cooled from 80 °C). All the intensities are Lorentz and multiplicity corrected.

(*hkl*)	experimental *d*-spacings (Å)	calculated *d*-spacings (Å)	*Intensity*
(400)[a]	46.7	46.7	100.0
(220)			93.0
(111)	37.4	37.4	0.3
(311)	32.5	32.5	0.5
(620)	27.0	27.0	1.4
(511)[b]	26.8	26.7	0.8
(131)		26.7	0.1
(800)	23.4	23.4	1.1
(440)		23.3	1.0
(711)[c]	21.9	21.9	0.3
(531)			0.3
a = 186.8 Å, *b* = 107.8 Å and *c* = 40.8 Å			

a: As (400) is on the meridian of the GISAXS pattern, its intensity cannot be accurately determined (sensitive to incident beam angle and distribution of domain orientations), consequently it is not possible to determine the (400) and (220) intensity ratio in the same way as described before for (511) and (620) peaks of IC[3]/12. It is assumed that the ratio of the two is the same as that of two in powder SAXS of IC[3]/12. Similar treatment is used to calculate the intensities of (800) and (440).

b: The ratio of the intensities of (511) and (131) from the GISAXS pattern can be calculated to be 0.85:0.15.

c: As the intensities of (711) and (531) are too weak to be observed in GISAXS pattern, the contribution of them in powder SAXS is assumed to be the same as in IC[3]/12.

FCN16

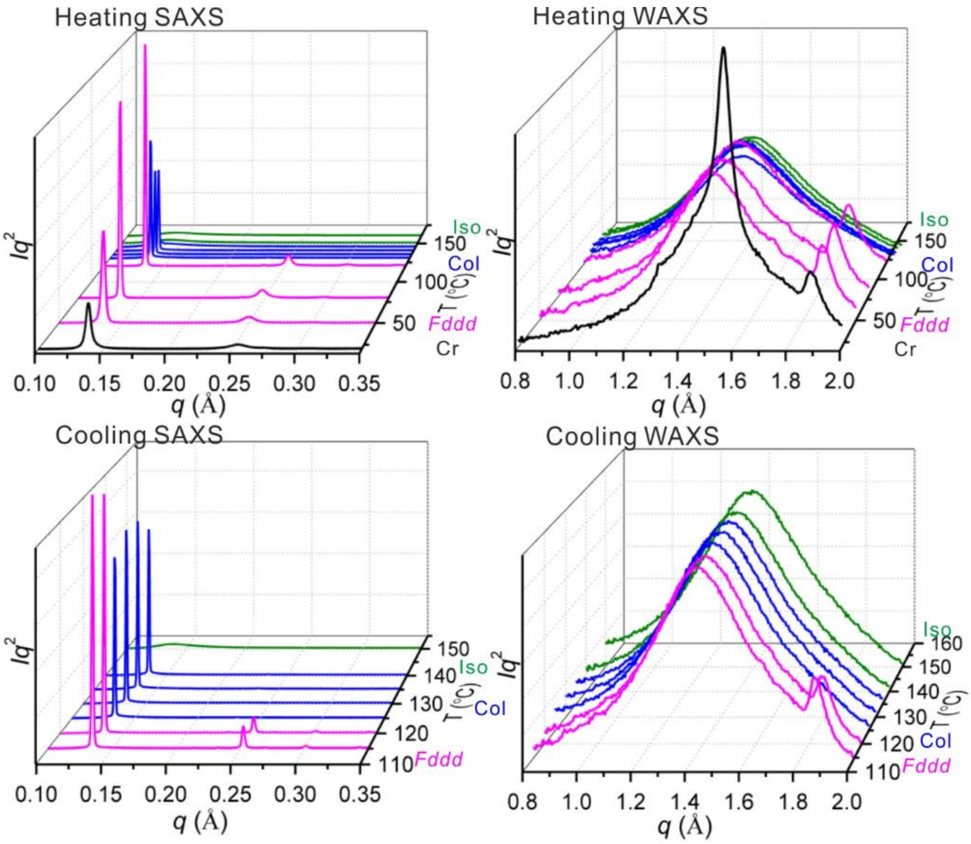

Supplementary Figure 7. SAXS/WAXS heating and cooling scans of FCN16.

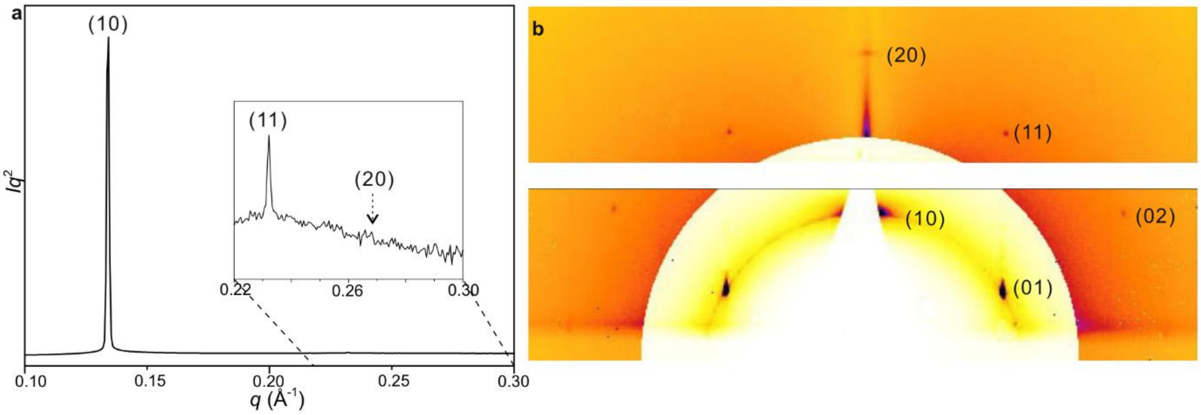

Supplementary Figure 8. a, Powder SAXS curve and **b**, GISAXS pattern of Col phase of FCN16 recorded at 130 °C (cooled from 150 °C). In SAXS curve, the intensity of (20) is too weak to be observed, as indicated by the arrow.

Supplementary Table 4. Indices, experimental, calculated *d*-spacings, intensities, phases and lattice parameters of diffraction peaks of FCN16 recorded by transmission powder SAXS in the Col phase at 130 °C (cooled from 150 °C). All the intensities are Lorentz and multiplicity corrected.

(*hk*)	experimental *d*-spacings (Å)	calculated *d*-spacings (Å)	Intensity	Phase
(10)	46.9	46.9	100.0	0
(11)	27.1	27.1	0.03	0
(20)	-	23.4	-	-
		a = 54.1 Å		

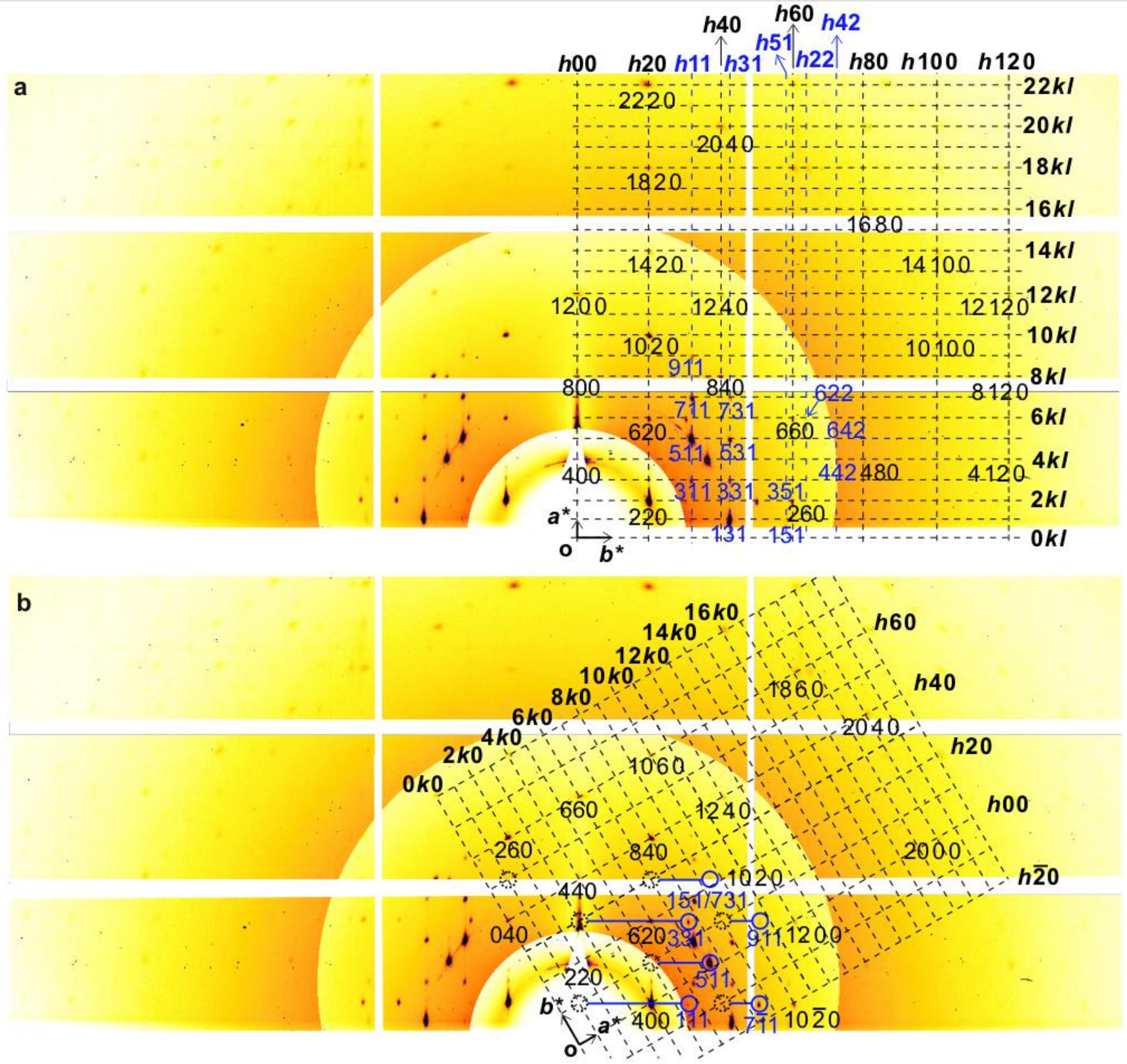

Supplementary Figure 9. a, **b** The GISAXS pattern of *Fddd* phase of FCN16 recorded at 120 °C (cooled from 150 °C). In b, some *hkl* spots are circled blue and connected to their *hk*0 base by blue row lines. The position of the projection of reflection (*hkl*) rotating around (110) on the 2D plane is calculated by $\left(\sqrt{\frac{h^2}{a^2}+\frac{l^2}{b^2}+\frac{l^2}{c^2}-\frac{(\frac{h}{a^2}+\frac{k}{b^2})^2}{\frac{1}{a^2}+\frac{1}{b^2}}}, \frac{\frac{h}{a^2}+\frac{k}{b^2}}{\sqrt{\frac{1}{a^2}+\frac{1}{b^2}}}\right)$.

Supplementary Table 5. Indices, experimental, calculated *d*-spacings, intensities, phases and lattice parameters of diffraction peaks of FCN16 recorded by transmission powder SAXS in the *Fddd* phase at 120 °C (cooled from 160 °C). All the intensities are Lorentz and multiplicity corrected.

(*hkl*)[a]	experimental *d*-spacings (Å)	calculated *d*-spacings (Å)	*Intensity*	*Phase*
(400)	46.7	46.7	100.0	π
(220)			93.0	π
(111)	32.7	32.7	0.1	-
(311)	29.2	29.3	0.2	-
(620)	27.0	27.0	0.4	-
(511)	24.8	24.8	8.4	π
(131)			1.5	π
(331)	23.3	23.2	0.2	-
(711)	20.8	20.8	0.6	π
(531)			0.6	0
(731)	18.3	18.3	0.2	-
(151)			0.2	-
a = 186.9 Å, *b* = 107.9 Å and *c* = 34.9 Å				

a: The calculation of the contribution of each reflection (*hkl*) to overlapped peaks in powder diffraction pattern was the same as in IC^3/12 and IC^3/14.

FO16

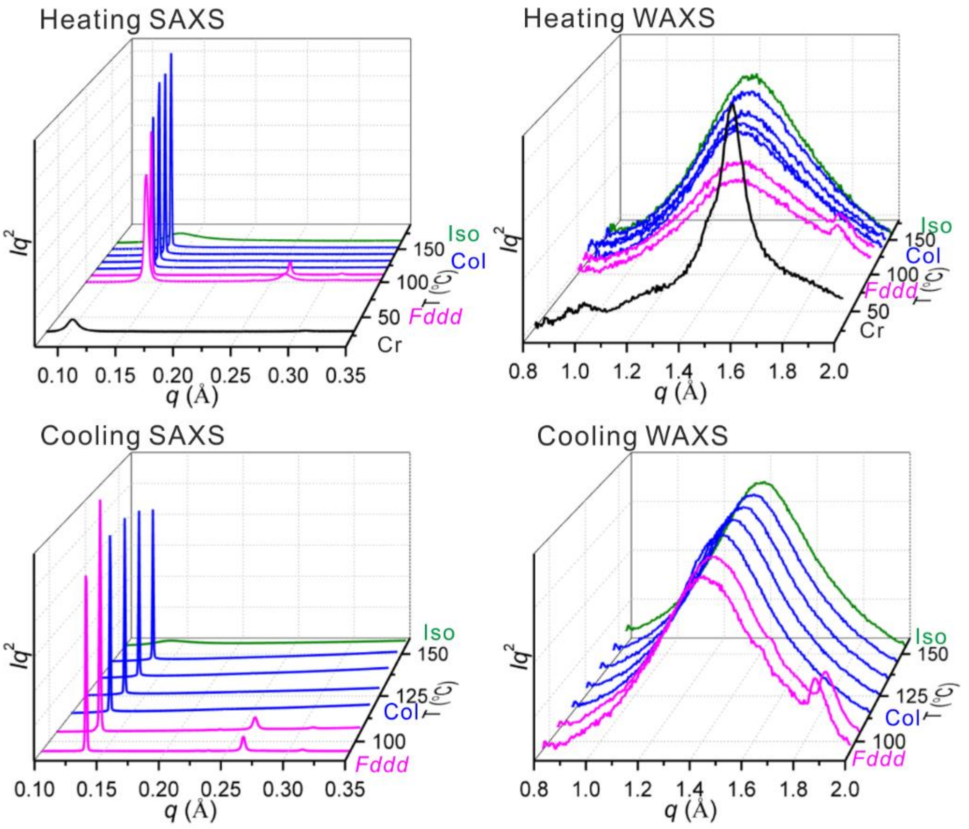

Supplementary Figure 10. SAXS/WAXS heating and cooling scans of FO16.

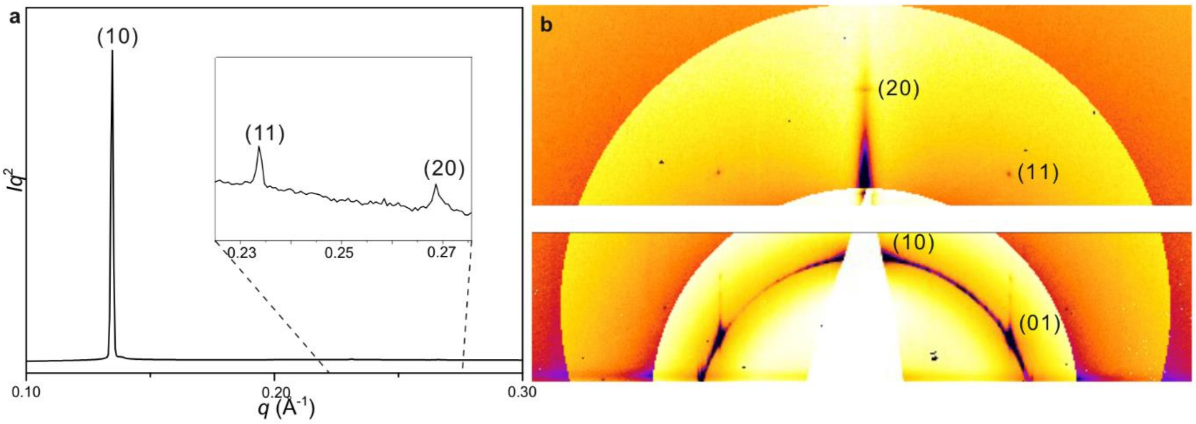

Supplementary Figure 11. **a**, Powder SAXS curve and **b**, GISAXS pattern of Col phase of FO16 recorded at 135 and 130°C, respectively (cooled from 150 °C).

Supplementary Table 6. Indices, experimental, calculated *d*-spacings, intensities, phases and lattice parameters of diffraction peaks of FO16 recorded by transmission powder SAXS in the Col phase at 135 °C (cooled from 150 °C). All the intensities are Lorentz and multiplicity corrected.

(*hk*)	experimental *d*-spacings (Å)	calculated *d*-spacings (Å)	*Intensity*	*Phase*
(10)	46.7	46.7	100	0
(11)	27.0	27.0	0.2	0
(20)	23.4	23.3	0.1	π
		a = 53.9 Å		

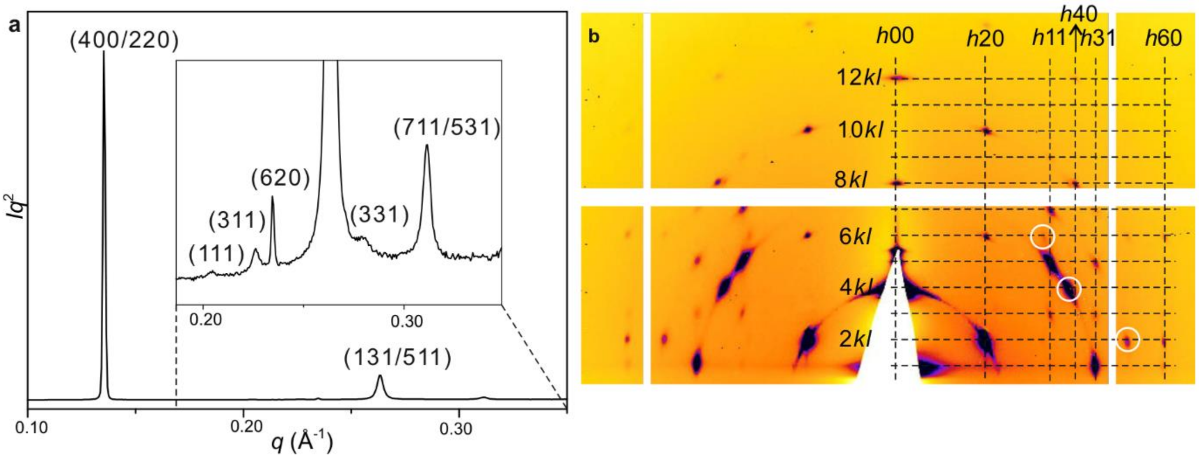

Supplementary Figure 12. **a**, Powder SAXS curve and **b**, GISAXS pattern of *Fddd* phase of FO16 recorded at 105 °C (cooled from 150 °C). The white circled spots are (511), (331) and (7$\bar{1}$1) from the other orientation.

Supplementary Table 7. Indices, experimental, calculated *d*-spacings, intensities, phases and lattice parameters of diffraction peaks of FO16 recorded by transmission powder SAXS in the *Fddd* phase at 105 °C (cooled from 150 °C). All the intensities are Lorentz and multiplicity corrected.

(*hkl*)[a]	experimental *d*-spacings (Å)	calculated *d*-spacings (Å)	*Intensity*
(400)<br>(220)	46.5	46.5	100.0<br>93.0
(111)	30.7	30.7	0.3
(311)	27.8	27.8	0.07
(620)	26.7	26.7	0.5
(511)<br>(131)	23.9	23.9	4.0<br>0.7
(331)	22.4	22.4	0.4
(711)<br>(531)	20.2	20.2	0.1<br>0.1
a = 186.0 Å, *b* = 107.4 Å and *c* = 32.5 Å			

a: The calculation of the contribution of each reflection (*hkl*) to overlapped peaks in the powder diffraction pattern was the same as in IC^3/12 and IC^3/14.

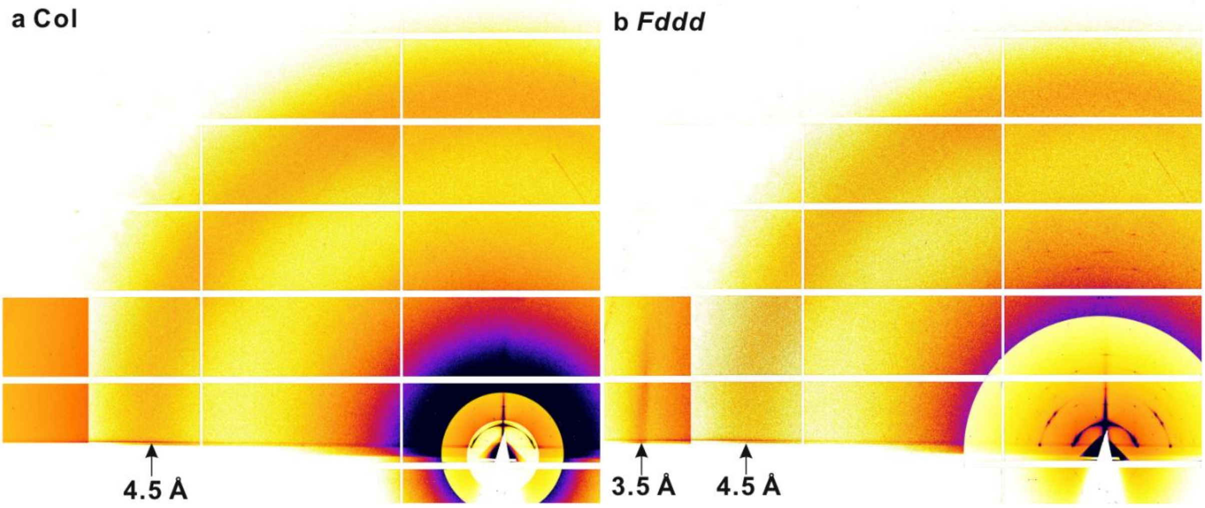

Supplementary Figure 13. GIWAXS/GISAXS patterns of **a** Col and **b** *Fddd* of FO16 recorded at 115 °C and 90 °C, respectively.

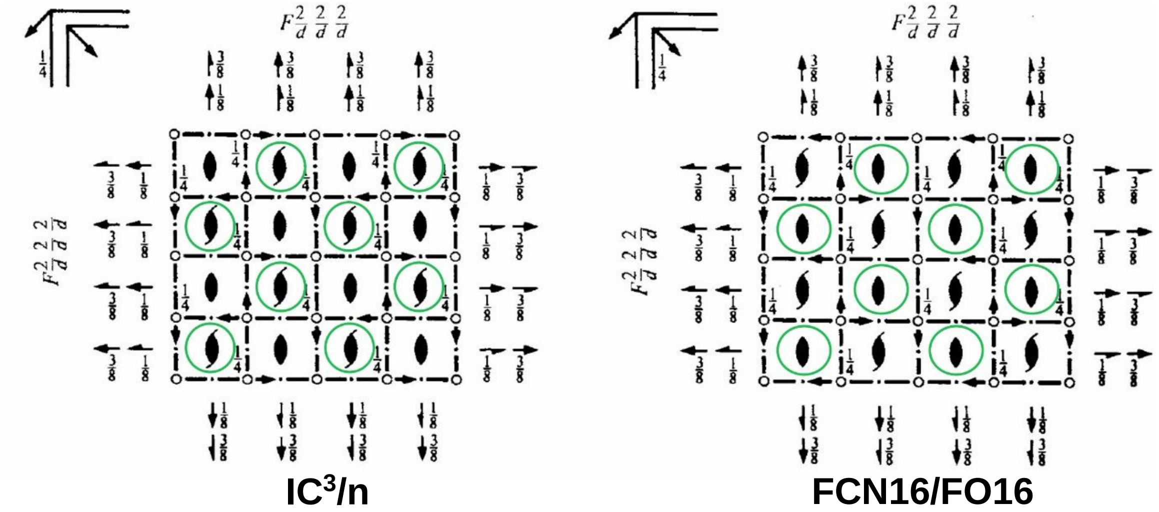

Supplementary Figure 14. Two alternative settings of the *Fddd* unit cell with all symmetry elements marked (from International Tables of Crystallography, Vol. A). The locations of columns are indicated with green circles: for the triangular twisted columns of IC^3/12 and IC^3/12 on 2_1 screw axes (left) and the ribbon-like twisted columns of FCN16 and FO16 on two-fold rotation axes (right).

Supplementary Section 4. Second harmonic generation

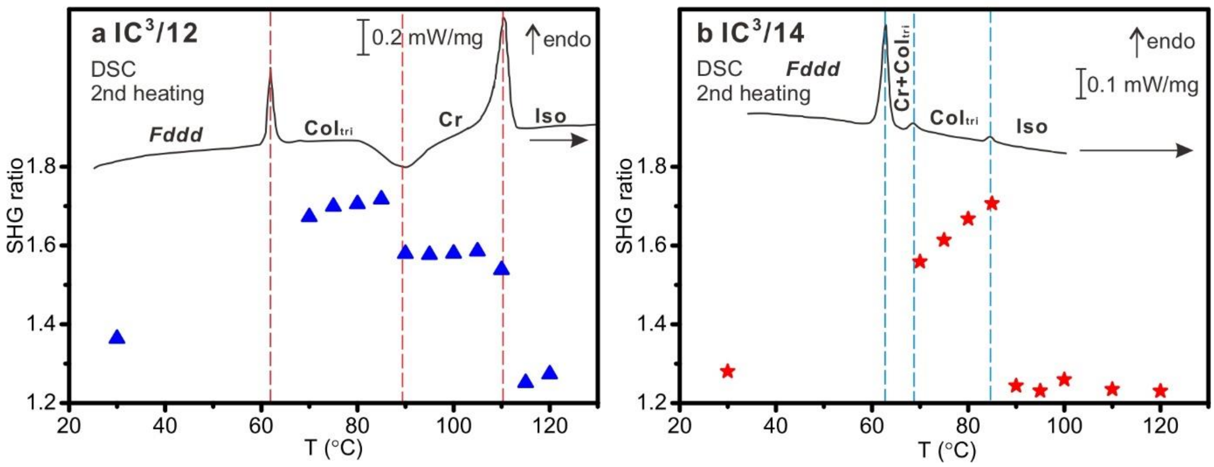

Supplementary Figure 15. Temperature dependence of intensity of the second harmonic (400 nm) vs. background for 800 nm excitation, generated 20 μm above the substrate surface in (**a**) IC^3/12 and **(b)** IC^3/14 recorded on heating. The residual SHG ratio of ca. 1.3 is due to surface effects – for more details see ref. 1. DSC curves are shown above to indicate the phase transitions.

Supplementary Section 5. Reconstruction of electron density maps

Starting with $IC^3/12$, its six strongest peaks (220), (400), (131), (511), (620) and (440) in Supplementary Table 1 are used to reconstruct the electron density (ED) map of the low-T phase based on *Fddd* symmetry. As *Fddd* is centrosymmetric, there are eight possible phase combinations as the phase of each peak can be only 0 or π. Some of these phase combinations are equivalent by simple shift of origin or full phase inversion. The structure should be closely related to the Col phase of the bent-core IC^3/n, judging by the negligible or small changes in position of the strongest diffraction peaks, POM texture, birefringence and transition enthalpy. The triangular cross-section of the columns thus precludes their positions on two-fold rotation axes of the *Fddd* unit cell (see Supplementary Fig. 14), which means that reflections (220) and (400) must have different phases. As the aromatic column cores have higher ED than the surrounding aliphatic continuum, the only remaining choices is 0 for (220) and π for (400). In contrast, the linear molecules of the FCN*n* compounds should be parallel to each other in a column stratum and their symmetrical nature implies that the columns have two-fold rotational symmetry. Therefore they lie along the 2-fold axes of the unit cell, as shown in Supplementary Fig. 14, meaning that the phase choice of reflections (220) and (400) must be π, π. The phases of other, weaker reflections is determined by trial and error with the main criterion being the match with the model of rotating strata at every *z*-level, as shown in Supplementary Figs. 17-18. A powerful endorsement of the choice is the match with the AFM images of the counter-rotating helices in Fig. 3j.

Supplementary Fig. 16 below show the 2D ED maps of the high-temperature columnar phase: hexagonal for FCN16 and FO16 and trigonal for $IC^3/12$ (see also ref. 1).

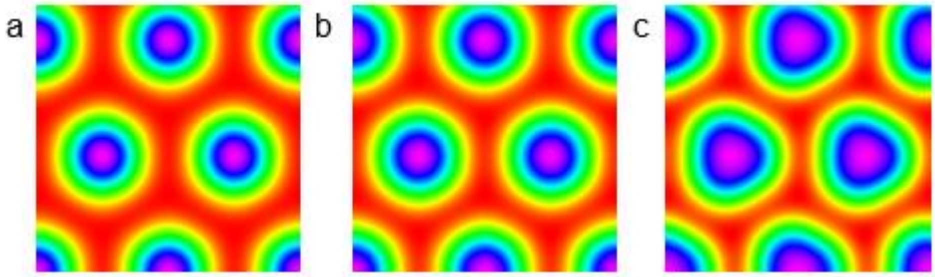

Supplementary Figure 16. ED maps of Col phases of **a** FCN16, **b** FO16 and **c** $IC^3/12$[1].

Supplementary Section 6. Estimate of the number of molecules per column stratum

Supplementary Table 8. Calculation of number of molecules per column stratum of *Fddd* and Col phases based on density. The results are independently verified by comparing the resulting molecular volumes with those calculated by the crystal increment method – see Supplementary Table 9.

Compound		IC^3/10	IC^3/12		IC^3/14		FCN16		FO16	
Phase		Col	*Fddd*	Col	*Fddd*	Col	*Fddd*	Col	*Fddd*	Col
Lattice parameter (Å)	*a*	48.9	173.5	51.3	186.8	52.8	186.9	54.1	186.0	53.9
	b	-	106.1	-	107.8	-	107.9	-	107.4	-
	c	-	40.8	-	40.8	-	34.9	-	32.5	-
Height of column stratum *h'* or *h* (Å)[a]		4.5	4.5	4.5	4.5	4.6	3.5 (*h*)	4.7	3.5 (*h*)	4.7
Volume of column stratum V_{strat} (Å^3/10^3)[b]		9.4	10.4	10.4	11.3	11.2	8.8	12.0	8.7	11.8
Number of molecules per stratum μ[c]		3.0	3.0	3.0	3.0	3.0	2.2	3.0	2.2	3.0

a: *h'* is the average intermolecular distance along the column axis (or stratum thickness) for Col phase of all compounds, as well as for *Fddd* phase of IC^3/n. By analogy with similar compounds, *h'* is estimated to be within 4.6 ± 0.1 Å, with a minor adjustment up to ±0.1 Å to make μ an integer. For *Fddd* phase of FCN16 and FO16, *h* is determined from the position of the meridional streak in GIWAXS.

b: Volume of a column stratum. *Fddd*: $V_{\mathrm{strat}} = abh/8$ or $abh'/8$; Col: $V_{strat} = a^2h' \sin 60°$.

c: Number of molecules per column stratum μ is calculated from $\rho = \mu M/N_A V_{strat}$, where ρ is density, *M* is molecular mass and N_A is Avogadro's number. Based on data on similar materials $\rho = 0.9$ g cm^{-3} is assumed. This choice is justified in detail in the Appendix at the end of this document.

Supplementary Table 9. Comparison of molecular volumes calculated by crystal volume increments and density methods.[a]

Name	IC^3/10	IC^3/12		IC^3/14		FCN16		FO16	
Phase	Col	*Fddd*	Col	*Fddd*	Col	*Fddd*	Col	*Fddd*	Col
V_{mol} by crystal increments (Å^3/10^3)[b]	2.99	3.39		3.78		3.82		3.76	
V_{mol} from density (Å^3/10^3)[c]	3.13	3.47	3.47	3.77	3.73	4.00	4.00	3.95	3.93

a: This table compares the molecular volume obtained independently by two different methods: (i) by crystal volume increments[2], whereby an incremental increase in volume by every atom is taken from an average of several hundreds or thousand of crystal structures, and (ii) from V_{strat} and μ values in Supplementary Table 8. The similarity of the obtained V_{mol} values provides further confidence in the numbers given in Supplementary Table 8.

b: Volume of the molecule: $V_{\mathrm{mol}} = V_{\mathrm{arom}} + V_{\mathrm{aliph}}$; V_{arom} = volume of aromatic part of the molecule calculated using the crystal volume increments[2]; V_{aliph} = volume of aliphatic part of the molecule assuming a density of 0.8 g cm^{-3} (density of liquid alkanes). The volume of the molecule in the next row is calculated by the density method and is shown for better comparison.

c: $V_{mol} = V_{strat}/\mu$

Supplementary Table 10. Twist angles between strata in *Fddd* phase.

Compound	$IC^3/12$	$IC^3/14$	FCN16	FO16
No. of strata per column per unit cell: $m = c/h^{(')}$	9.1	9.1	10.0	9.3
Twist angles between strata<br>IC^3/n: $\alpha = 120°/m$<br>FCN16/FO16: $\alpha = 180°/m$	13.2	13.2	18.0	19.4

Supplementary Table 11. Comparison of lattice parameters ratios for compounds in this work and block copolymers.

Compound name[a]	*c*:*b*:*a*
Block copolymers[3]	0.500 : 1 : √3
$IC^3/12$	0.385 : 1 : 1.635
$IC^3/14$	0.378 : 1 : √3
FCN16	0.323 : 1 : √3
FO16	0.298 : 1 : √3

a: Lattice parameter *a* is defined as the largest one.

Supplementary Section 7. Additional schematic models with ED maps

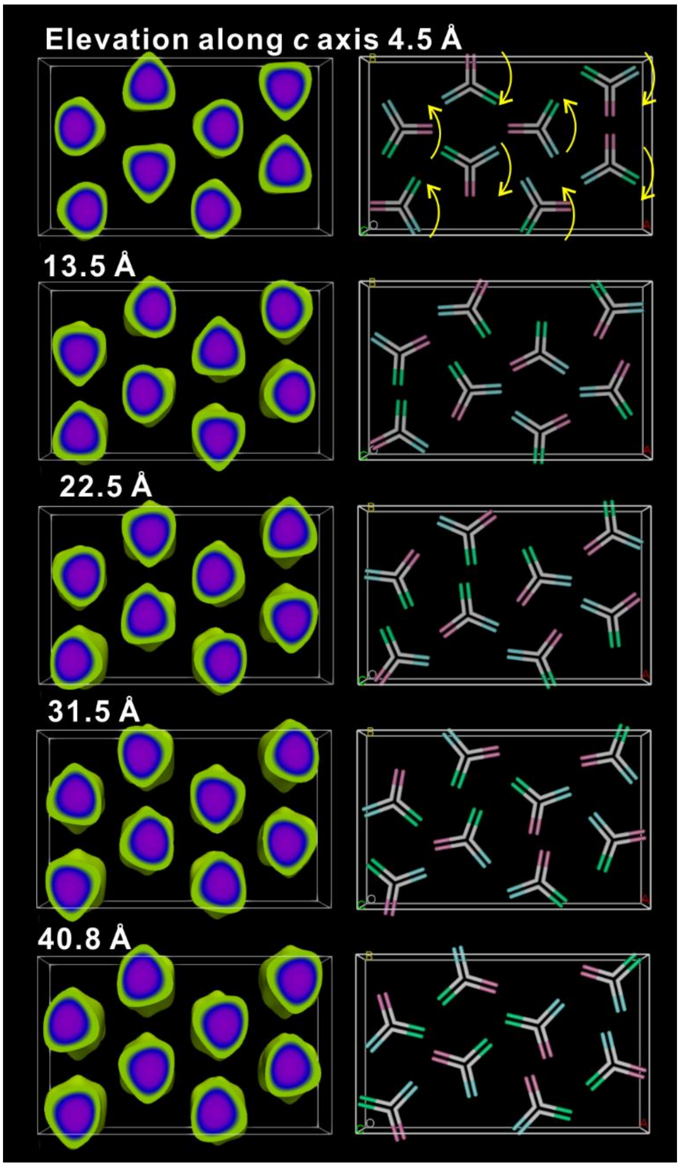

Supplementary Figure 17. Detailed ED maps (high ED region) and sketched models viewing along *c*-axis with different layers of $IC^3/12$ (see also videos 1.1-1.3).

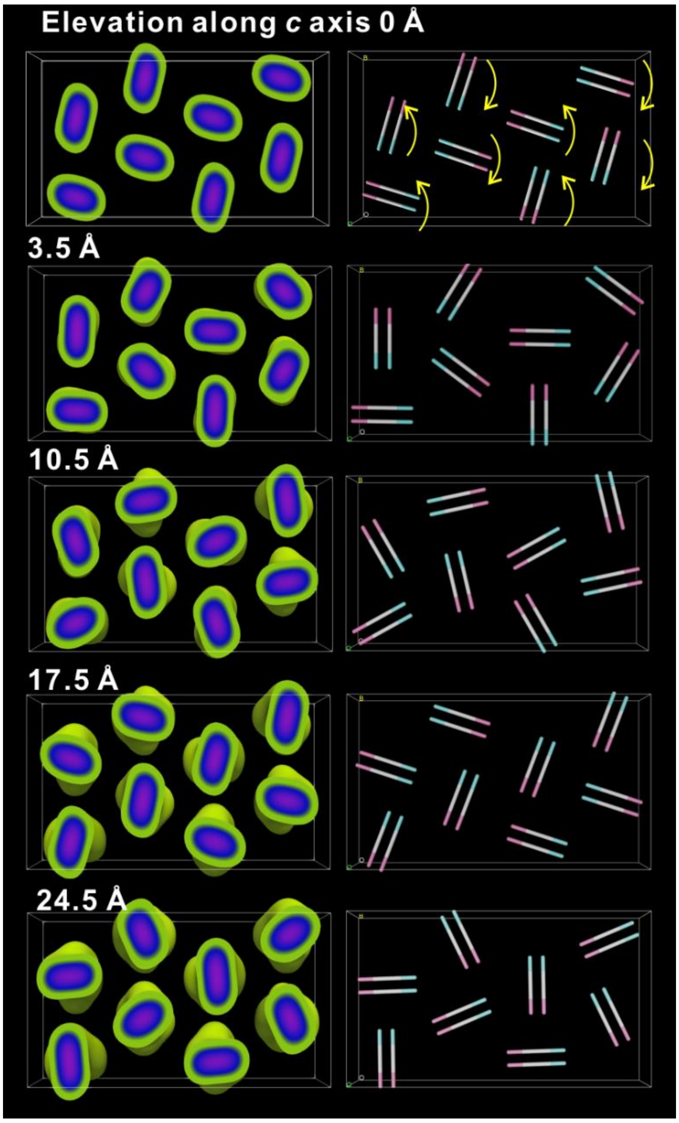

Supplementary Figure 18 Detailed ED maps (high ED region) and sketched models viewing along *c*-axis with different layers of FCN16 (see also videos 2.1-2.3).

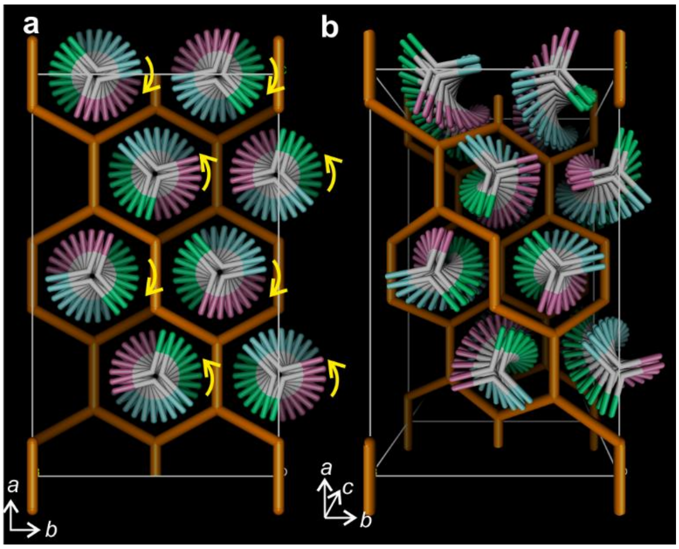

Supplementary Figure 19. Additional schematic *Fddd* models, comparing the single network model formed by block copolymers with the model of IC^3/n.

Supplementary Section 8. Additional AFM images

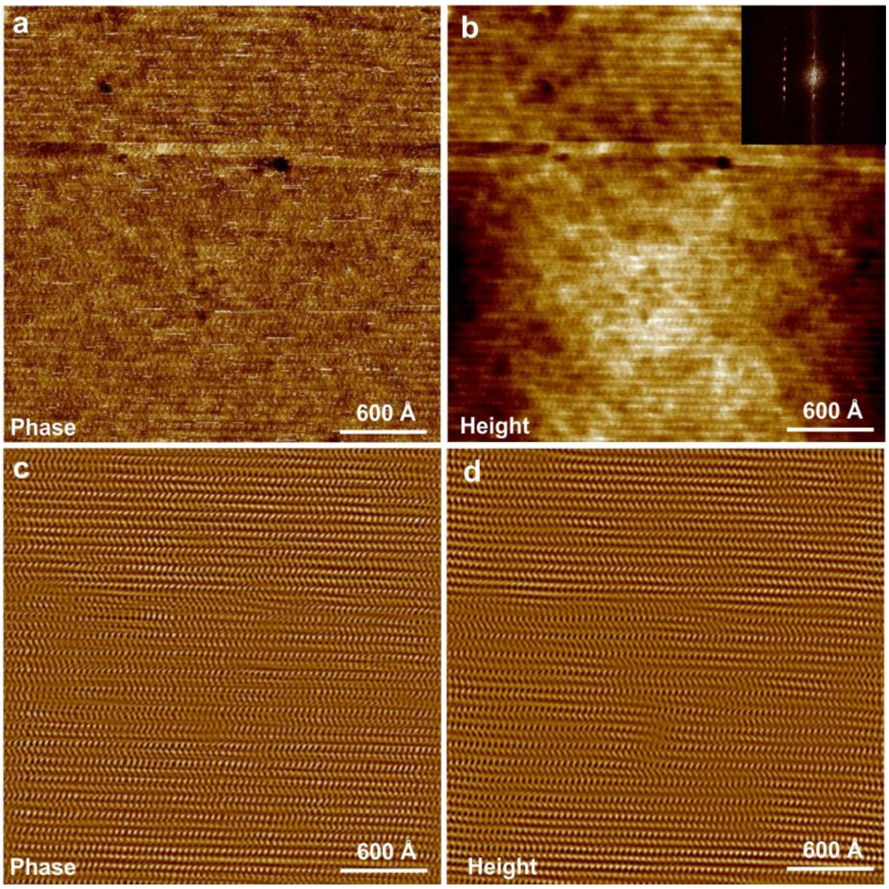

Supplementary Figure 20. AFM of FCN16 in the *Fddd phase* at 50°C: a, **b** original phase and height images; **c**, **d** corresponding Fourier filtered images. Inset in (**b**) shows the Fourier transform of (**b**).

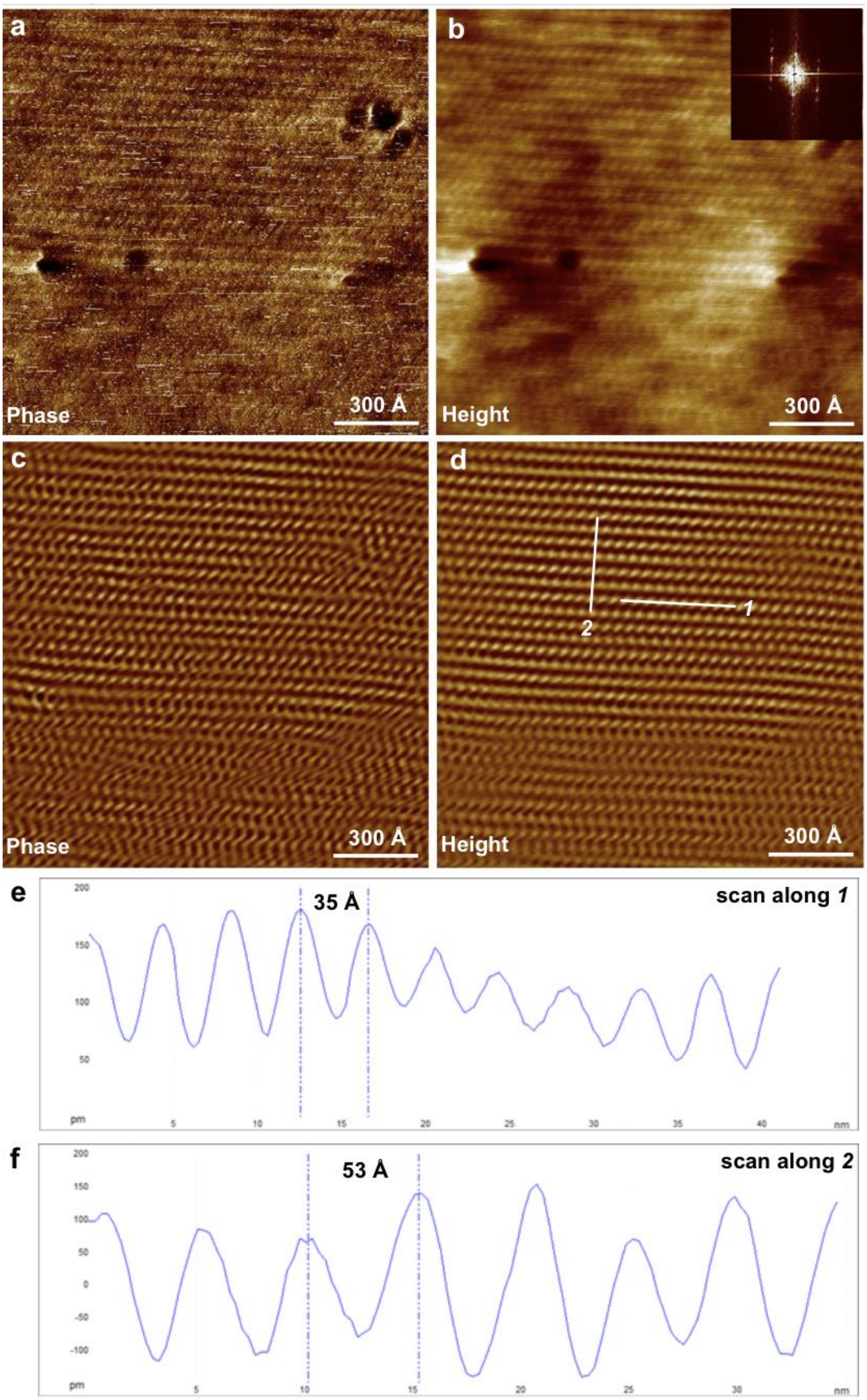

Supplementary Figure 21. AFM of FCN16 in the *Fddd phase* at 50°C at higher magnification: a, **b** original phase and height images; **c**, **d** corresponding Fourier filtered images; **e**, **f** scanned spectra along the two axes of (**d**).

Supplementary Section 9. UV-vis and fluorescence emission spectroscopy

Supplementary Table 12. UV-vis and Fluorescence Emission results

Compound	phase	Absorption λ_{max}/nm	Band-gap/eV[a]	Emission λ_{max}/nm[b]	Stokes shift (nm)
FCN16	Col	209, 396, 652	1.52 (130 °C)	-	-
FCN16	*Fddd*	209, 412, 696-714	1.42 (120 °C)<br>1.35 (30 °C)	-	-
FO16	Col	209, 316, 384, ~500	2.15 (110 °C)	625-633	113-121
FO16	*Fddd*	211, 321, 396, ~520	2.13 (100 °C)<br>2.10 (40 °C)	642-646	130

a: Band-gap, based on the longest wavelength absorption, is calculated by the Tauc plot method[4]. The band-gap of FCN16 is smaller than that of FO16 due to the stronger electron-accepting ability of the fluoren-9-ylidene malononitrile group.

b: λ_{max} in Emission spectra.

Supplementary Discussion 1. Minimum energy packing of helical columns

We have developed a simple quantitative theory in order to examine the interactions and packings of helical columns, and to explore their possible minimum energy structures. The theory suggests that the *Fddd* structure is indeed the minimum energy structure from packing of helical columns, among the different candidate structures we have explored.

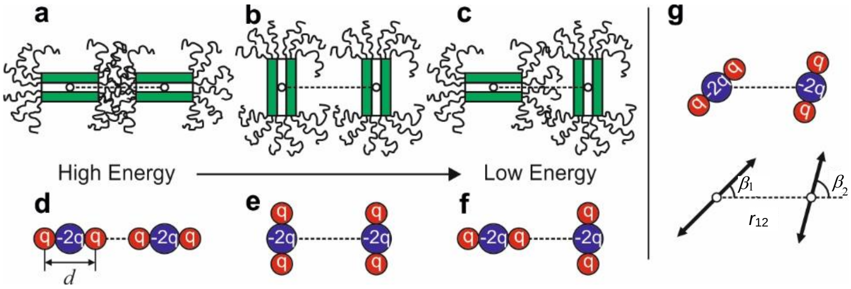

Supplementary Figure 22. (a-c) Different orientations of two dimers of straight core phasmids in neighbouring columns result in different system energies. (a) Highest energy state due to heavy clashes between aliphatic chain ends of the dimers. (b) Medium energy state due to inefficient packing of space (voids between straight cores of the dimers). (c) Minimum energy state where the space is efficiently packed with little clashes between chain ends. Such energy landscape matches that from the interaction of two linear quadrupoles, with the orientations of the quadrupoles the same as those of the dimers (d-f). (g) The interaction energy between two linear quadrupoles are linked to their distance r_{12} and their orientations as defined by angles β_1 and β_2.

In FCN16 and FO16, the basic unit of the helical column is the dimer. In each stratum of the helical column there are two molecules, with their straight cores parallel to each other and six flexible aliphatic chains at each ends of the dimer core (Fig. 4**d**). As shown in Supplementary Fig. 22a-c, how efficiently the space is filled between two columns very much depends on the orientations of the dimers in two neighbouring columns. The interaction energy between the two dimers is high if they are pointing at each other as their chain ends would clash. There is less clash between two dimers if they are pointing away from each other (Supplementary Fig. 22b), but the voids between the two dimers means inefficiency in space packing hence it is high energy too. The best packing of space is achieved when one dimer is pointing directly at the other, while the other is pointed way (oriented perpendicular to the other), as in Supplementary Fig. 22**c**. Interestingly, such dependence of interaction energy between the dimers on their orientations is very much similar to that between two linear quadrupoles, as shown in Supplementary Figure 1d-f, the energy is highest when the two quadrupoles are pointing at each other, lowest when one of them points at another, while the other points perpendicularly away.

The interaction energy between two quadrupoles, with quadrupole moment $\phi = 3qd^2$ (*q* is the partial charge at the ends and *d* is the length of the quadrupole, as shown in Supplementary Fig. 22d), is

$$E_{\phi-\phi} = A\frac{\phi^2}{r_{12}^5}$$

Where r_{12} is the distance between the two quadruples and *A* is the factor linked to the orientations of the two quadrupoles. In our case the two quadrupoles are in the same plane and their orientations are defined by two angles β_1 and β_2 (Supplementary Fig. 22g)[5].

$$A = 3\cos(2\beta_1 + 2\beta_2) + \frac{9}{16}\cos 2\beta_1 \cos 2\beta_2 + \frac{15}{16}\cos 2\beta_1 + \frac{15}{16}\cos 2\beta_2 + \frac{9}{16}$$

The minimum values of A is found with $\beta_1 = 0$ and $\beta_2 = \pi/2$ (or $\beta_1 = \pi/2$ and $\beta_2 = 0$), with $A_{min} = -3$. The maximum value of A is found with $\beta_1 = \beta_2 = 0$, with $A_{max} = 6$. For random arrangement of β_1 and β_2, the averaged A is $A_{ave} = 9/16$.

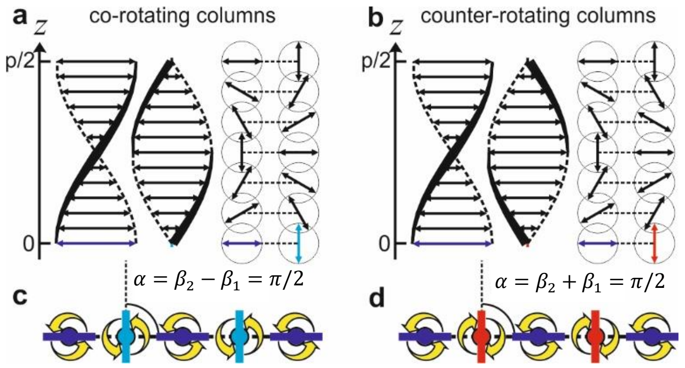

Supplementary Figure 23. (a, b) Side and projected views of minimum energy neighbouring co-rotating (a) and counter-rotating (b) helical columns. Twisting of dimers along the columnar direction (*z*-axis) is represented as a series of twisting linear quadrupoles. (c,d) The minimum energy 1D array of helical columns, with nearest neighbours always co-rotating (c) or counter-rotating (d). The rotating direction of each column is shown by yellow arrows, and the orientation of the quadrupole at z=0 is shown by the coloured rod at the centre. The interaction between two neighbouring co-rotating and counter-rotating helices depends on an angle α. For co-rotating helical columns $\alpha = \beta_2 - \beta_1$, and for counter-rotating ones $\alpha = \beta_1 + \beta_2$. Here β_1 and β_2 define the orientations of the two quadrupoles, one from each column, at the same *z*-level. α is a constant independent of *z* and defines the relative orientation of the two helical columns.

To calculate the interaction energy of two helical columns, each of which consists of a series of dimers represented by quadrupoles, with the direction of dimer/quadrupoles twisting along its helical axis (Supplementary Fig. 23a,b), we make the assumption that the helix is continuous, and the interaction between the two helical columns is simply the average of quadrupole interactions at different heights, i.e. we ignore the interactions between dimers from the two columns at different heights. This, even though much simplified, is in fact equivalent to considering the average orientation of dimers/quadrupoles that are interacting with a dimer/quadrupole from another column, and should carry the essence of such interaction between helical columns.

If the two neighbouring columns are co-rotating, i.e. have the same hand, then at different height we always have $\alpha = \beta_2 - \beta_1$ being a constant (Supplementary Fig. 23a). When the height *z* goes from 0 to *p*/2, where *p* is the helical pitch of the column, $\beta_1$changes from 0 to π and β_2 changes from α to $\alpha + \pi$. Average at different heights thus gives the interaction energy per pair of dimers between co-rotating columns as

$$U_{co} = \frac{2\phi^2}{pr_{12}^5}\int_0^{p/2} A_{co}(z)dz = \frac{3\phi^2}{32r_{12}^5}(3\cos 2\alpha + 6)$$

If the two neighbouring columns are counter rotating instead, at different height $\alpha = \beta_1 + \beta_2$is a constant (Supplementary Fig. 22b). When the height *z* goes from 0 to *p*/2, $\beta_1$changes from 0 to π and β_2 changes from α to $\alpha - \pi$. Average at different heights thus gives

$$U_{counter} = \frac{2\phi^2}{pr_{12}^5}\int_0^{p/2} A_{counter}(z)dz = \frac{3\phi^2}{32r_{12}^5}(35\cos 2\alpha + 6)$$

In both cases the energy minimum is given by $\alpha = \pi/2$, i.e. when at a particular height the dimer from one column is pointing at another column, while the dimer in the other column at the same height is pointing perpendicularly away. It is also obvious from the calculation that neighbouring counter-rotating helices is more favoured energetically comparing to co-rotating ones. Such behaviour is like that of two intermeshing cogwheels, where they rotate in opposite directions so the teeth of each cogwheel always avoid direct clash with those of another.

The minimum energy configuration of our helical columns can be readily derived in 1D, as all neighbouring columns can be counter rotating and with α angle being $\pi/2$, as shown in Supplementary Fig. 23d. If all the columns have the same handedness hence co-rotating, the 1D minimum energy configuration is shown in Supplementary Fig. 23**c**, again with all α angles being $\pi/2$. Expressing the energy in the unit of $\frac{3\phi^2}{32r_{12}^5}$, the minimum energy per dimer in a 1D counter-rotating array is -29, that of the co-rotating array is 3, and for a random 1D array (without any *z*-correlation between columns) the energy per dimer is 6.

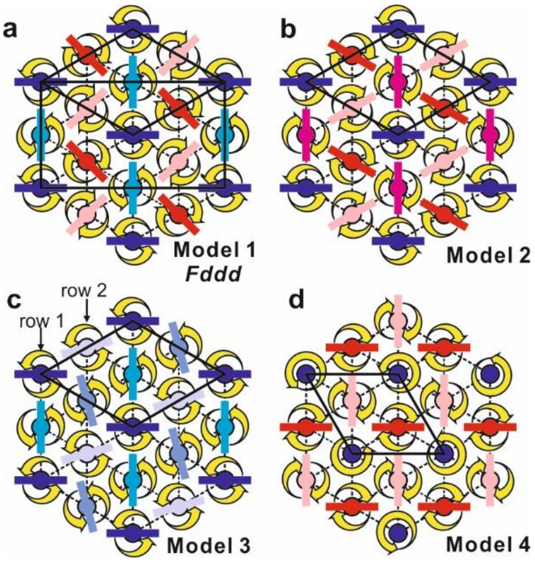

Supplementary Figure 24. (a) Calculated minimum energy configuration on a 2D hexagonal lattice, with two left- and two right-handed columns in a 2x2 hexagonal unit cell. The result is equivalent to the *Fddd* structure we have observed experimentally. (b) Calculated minimum energy configuration on a 2D hexagonal lattice, with three left- and one right-handed columns in a 2x2 hexagonal unit cell. (c) Calculated minimum energy configuration on a 2D hexagonal lattice, with four right-handed columns in a 2x2 hexagonal unit cell. It turns out that vertical shifting of the second row of columns, in relation to the first row, does not change the system energy. (d) Calculated minimum energy configuration on a 2D hexagonal lattice, with two left- and one right-handed columns in a $\sqrt{3} \times \sqrt{3}$ hexagonal unit cell. The orientation of the right-handed (red) columns can be random without affecting the system energy.

However, the condition to keep all neighbouring columns counter-rotating cannot be satisfied on a 2D hexagonal lattice, as around each triangle in the lattice there are three columns neighbouring to each other, and at least two of them must have the same hand. The obvious choice to minimize the system energy is to keep as many counter-rotating neighbours for each column as possible. As shown in Supplementary Fig. 24a, a 2x2 periodic supercell is assumed, as both counter- and co-rotating minimum energy 1D columns have a repetition every two columns (with α angle being $\pi/2$). We have two left-and two right-handed helical columns in the unit cell, and each column has four counter rotating and two co-rotating neighbouring columns (a ratio of 2:1). It is also not possible to make all rows of co-rotating and counter-rotating columns to have α angles of $\pi/2$. Energy minimization shows that all co-rotating

columns have αangles of $\pi/2$, and all counter-rotating columns have an α angle of $5\alpha/12$ (Supplementary Fig. 24a), with an average energy per dimer of about –45.6 (in unit of $\frac{3\phi^2}{32r_{12}^5}$, Supplementary Table 13). The minimum energy configuration fits very well with our experimental observations (Fig. 4b in the main text).

While the model shown in Supplementary Fig. 24a seems reasonable, we have also explored other possible configurations on a 2D lattice and these are shown in Supplementary Fig. 24b-d. In the configuration in Supplementary Fig. 24b, we have again a 2x2 superlattice, but with three left-handed and one right-handed columns. While around a right-handed column, all its six neighbouring columns are counter-rotating, for the left-handed columns, four of its neighbouring columns are co-rotating and the other two are counter-rotating. Overall the ratio between counter-rotating neighbours to co-rotating ones is 1:1. While the α angles between counter-rotating columns are $\pi/2$, the α angles between co-rotating columns are $\pi/3$. Due to the reduced number of neighbouring counter-rotating columns, it is no surprise that this structure turns out to have a higher energy than our *Fddd* structure (-36.75 vs -46.5 per dimer in unit of $\frac{3\phi^2}{32r_{12}^5}$, Supplementary Table 13).

When all columns have the same handedness, as shown in Supplementary Fig. 24c, the energy is found to be much higher (15 in unit of $\frac{3\phi^2}{32r_{12}^5}$). There are also many equivalent minimum energy configurations. While row 1 and row 2, as shown in Supplementary Figure 3e, have α angles of $\pi/2$, the system energy will not be affected by a change in the relative angles (or equivalent shift of the rows in the *z*-direction) between the two rows.

The other configuration we have studied is shown in Supplementary Fig. 24d, a three-column super-lattice structure similar to that proposed for the so-called "ordered columnar hexagonal" phase found in temperatures below the normal discotic columnar hexagonal phase in Hexa-hexylthiotriphenylene (HHTT)[6]. It has three columns in its unit cell, two left-handed and one-right handed. In its calculated minimum energy configuration, all neighbouring co-rotating columns have α angle of $\pi/2$. The system energy, however, does not depend on the orientation of the right-handed columns at all. The calculated energy turns out to be exactly the same as that of the previous model but much worse than the first two, where rows of counter-rotating columns have been better preserved.

In summary, our theoretical calculations strongly indicate that the *Fddd* structure we have observed is the direct result of optimizing the packing efficiency, and therefore the best structural candidate, of helical columns.

Supplementary Table 13. Calculated minimum interaction energies for different regular configurations of helical columns on a hexagonal lattice, given as average energy per dimer. For comparison, a columnar hexagonal phase of helical columns without any correlation in the *z*-direction between columns has an average energy per column of 18 (in unit of $\frac{3\phi^2}{32r_{12}^5}$).

Model Number	1 (*Fddd*)	2	3	4
Energy per dimer (in $\frac{3\phi^2}{32r_{12}^5}$)	$15-35\sqrt{3}$ (~–45.6)	-36.75	15	15

Supplementary Discussion 2: Comments on the number of molecules per column stratum

Here we give some comments on our calculation of the number of molecules per column stratum μ, the results of which are given in Supplementary Tables 8 and 9.

There are two problems that may arise in the determination of μ in a columnar liquid crystal: (i) unknown thickness of the stratum and (ii) unknown density. Regarding (i), we have been fortunate in this work that compounds FCN16 and FO16 give a very clear diffraction streak at 3.4-3.5 Å in the *Fddd* phase in the GIXRD (see e.g. Fig. 2g,h,j), and in the powder pattern (Fig 2e). This diffraction peak is completely absent in the high-T columnar Col_{hex} phase (Fig 2e and f), as well as in both phases of the IC^3 compounds. As is well known, there are two main ways of stacking of benzene rings in crystals or liquid crystals: the T-contact, where rings on adjacent molecules are at a high angle to each other, mostly close to 90°, with the hydrogen on one ring pointing at the electron-rich centre of the other ring. In the alternative parallel contact case, the ring planes are parallel but the rings are shifted in-plane. The latter gives the diffraction peak in the 3.4-3.7Å region in columnar LCs, while the former only contributes to the diffuse scattering in the 4-5 Å range, non-distinct from the scattering on alkyl chains. In the former case (T-stack) the rings are inclined to the column axis, typically by ~45°, but the angle is somewhat variable resulting in a variable stratum thickness. The latter (parallel, or "π-π stacking") is common in molecules with condensed aromatic rings (discotics), giving them a narrow bandgap and semiconducting properties.

Our FCN and FO molecules have a fluorene-based group which is planar and could be thought of as containing three fused aromatic rings. The molecule could be considered as being borderline discotic. The XRD/GIXRD results (absence and presence of the 3.5Å diffraction) indicate a switch between the T-contact (Col_{hex}) and parallel stacking mode (*Fddd*) at the phase transition. Consistent with this switch is the spectroscopic behaviour, with significantly reduced bandgap and red shift upon the Col_{hex}-*Fddd* transition (Fig. 6). Associated with the T-stack → parallel-stack change is the drop in μ from 3 to ~2. The fact that the bent-core IC^3 compounds do not show a diffraction peak in the 3.5Å region is consistent with the above picture. They contain no fused plate-like moiety and thus their stacking is most likely T-type in both Col_{tri} and *Fddd* phases.

Regarding macroscopic density, point (ii) above, while sometimes this is measured at room temperature, it is very rarely measured at elevated temperatures where most LC phases occur. Dilatometry has however been used occasionally (e.g. by the Guillon-Donnio group). Without such direct measurement, relating the density of a high-T LC phase to the room-temperature density may be deceptive, due to high thermal expansion of LCs.

In the case of IC^3 compounds the 3-arm star electron density profile of the column is a direct proof of the stratum containing three molecules. In the case of pizza-slice-shaped tapered dendrons or minidendrons the column diameter mainly shows a continuous decrease with increasing temperature,[7] as expanding molecules are continuously ejected from the column on heating. In such cases only an average number of molecules per column segment can be given. In the present case of FCN16 and FO16 compounds in the *Fddd* phase the non-integer μ = 2.2 could be interpreted as meaning that 10% of strata contain 3 molecules. Interestingly, even in tapered minidendrons there are cases where the stratum contains a clear constant integer number of molecules as in Li[8,9] and Na[9] salts of 3,4,5-(dodecyloxy)benzoic acid. Even a quantized molecular ejection phase change from μ = 4.0 to 3.5 has been shown to occur through a 1st-order transition on heating the Na salt. In cases where μ is a temperature-independent known integer and where stratum thickness can be directly measured by XRD, crystallographic density and thermal expansivity can be measured fairly reliably. Thus for the above Na benzoate the density extrapolated to 120°C is 0.74 g cm^{-3}. 120°C is the temperature at which we determined μ in FCN16 in the current work, assuming a density of 0.9 g cm^{-3}. The fact that this density is higher than that of the benzoate is justified by the higher aromatic

fraction in the FCN/FO compounds. We also note that the molecular volume calculated using this density matches very well that calculated independently by the method of crystal increments[2], as shown in Supplementary Table 9. Also note that using the same density of 0.9 g cm^{-3}, the calculated μ in IC[3] compounds is exactly 3 when applying a value of 4.6 $\pm$ 0.1 Å for the stratum thickness.

Supplementary Methods. Synthesis and analytical data

1. General remarks

The synthesis of bent phasmid mesogens IC^3/10, IC^3/12 and IC^3/14 have been synthesized by a Cu(I)-catalyzed click reaction[1]. The compounds FO16 and FCN16 were synthesized using Suzuki coupling reactions as key step as shown in Scheme 1. Firstly, 2,7-dibromo-9H-fluorenone **3**[10] was obtained by the bromination and oxidation of fluorene **1**. Then 2,7-bis(4,4,5,5-tetramethyl-1,3,2-dioxaborolanyl)-9-fluorenone **4**[11] was obtained by the $PdCl_2$(dppf)-catalyzed reaction of the compound **3** with bis(pinacolato)diboron. 2-(4-((3,4,5-Tris(hexadecyloxy)benzyl)oxy)phenyl)thiophene **5** was synthesized according to our previously reported procedures[12]. **5** was brominated with N-bromosuccinimide (NBS) and the obtained 2-bromo-5-(4((3,4,5-tris(hexadecyloxy)benzyl)oxy)phenyl)thiophene **6** was coupled with compound **4** to afford compound FO16. Finally, a Knoevenagel condensation between malononitrile and FO16 led to the target product FCN16.

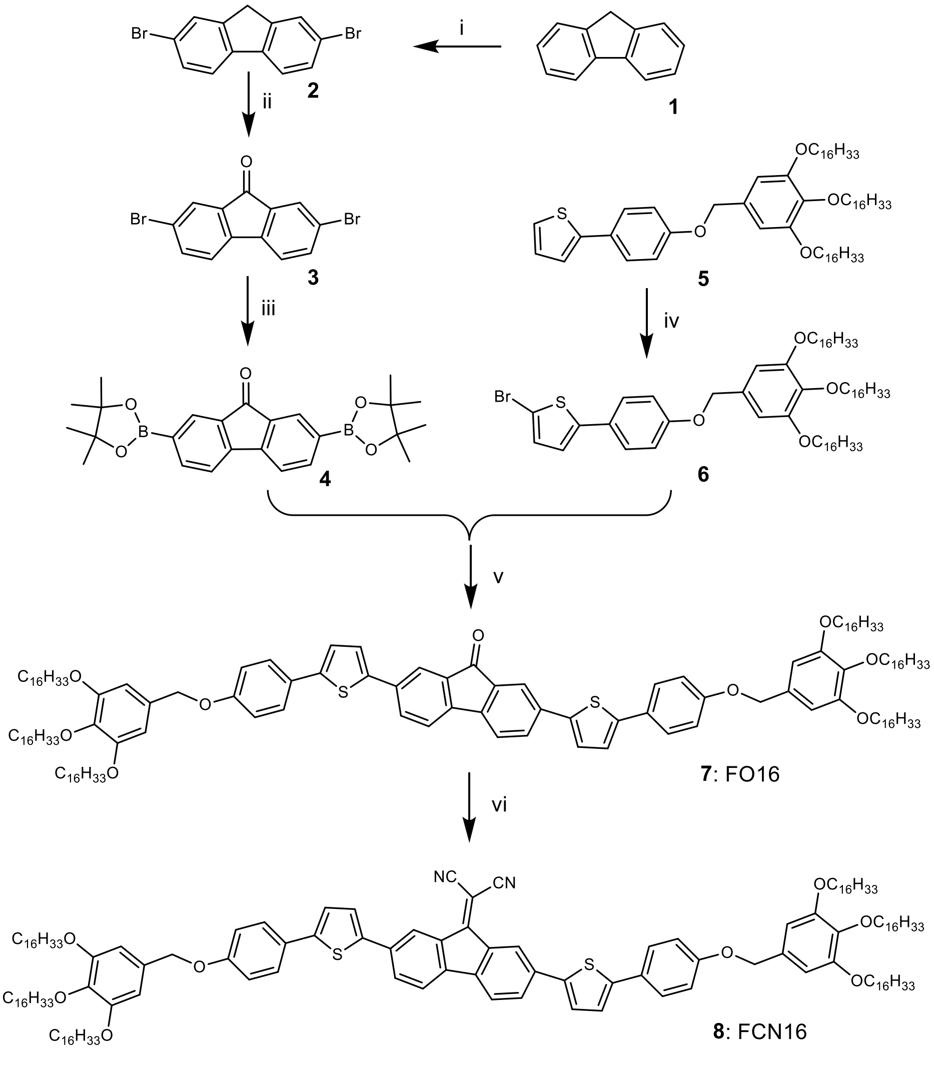

Supplementary Figure 25. Synthesis of compound FO16 and FCN16. Reagents and conditions: *i*) Br_2, $FeCl_3$, $CHCl_3$, 0 °C; *ii*) CrO_3, CH_3COOH, RT; *iii*) bis(pinacolato)diboron, $PdCl_2$(dppf), KOAc, 1,4-dioxane, 100 °C; *iv*) NBS, THF, 0 °C; *v*) $Pd(PPh_3)_4$, K_2CO_3, THF, H_2O, N_2, 78 °C; *vi*) $CH_2(CN)_2$, pyridine.

2. General procedures for the synthesis of FCN16.

Compound **2**:

Fluorene **1** (500 mg, 3.00 mmol) was dissolved in chloroform (5 mL) and $FeCl_3$ (7.5 mg, 0.046 mmol) was added. The solution was cooled in a water/ice bath to 0 °C. Bromine (0.33 mL, 6.34 mmol) was added dropwise to the stirred mixture. After complete addition the mixture was stirred for an additional three hours. Saturated $Na_2S_2O_3$ solution (20 mL) was slowly added and stirring was continued for 30 minutes. Chloroform (50 mL) was added, the organic phases were separated and the aqueous layer was extracted with chloroform (3 × 50 mL). The combined organic layers were dried over anhydrous Na_2SO_4, filtered and the solvent was evaporated in vacuo. The crude product was purified by a silicagel column chromatography (Petroleum ether) to produce compound **2** as white solid. Yield: 961.8 mg (98.7%). ^{1}H-NMR (300 MHz, $CDCl_3$), *δ* (ppm): 7.64-7.62 (d, *J* = 1.6 Hz, 2H, Ar**H**), 7.58-7.52 (t, *J* = 9.0 Hz, 2H, Ar**H**), 7.49-7.46 (d, *J* = 8.1 Hz, 2H, Ar**H**), 3.84-3.78 (d, *J* = 15.6 Hz, 2H, fluorene-9**H**). Elemental analysis calcd (%) for $C_{13}H_8Br_2$ (324.02): C, 48.19; H, 2.49; found: C, 48.37; H, 2.66.

Compound **3**:

A mixture of 2,7-dibromofluorene (896 mg, 2.7 mmol) and CrO_3 (6.00 g, 60.0 mmol) suspended in 25 mL acetic acid, and stirred at room temperature for 12 h. The resulting yellow precipitate was collected by suction filtration, washed with water thoroughly, and dried under vacuum to provide the product as yellow solid. The crude product was purified by a silicagel column chromatography (Petroleum ether) to produce compound **3** as white solid. Yield: 355 mg (37.9%). ^{1}H-NMR (400 MHz, $CDCl_3$), *δ* (ppm): 7.78 (s, 2H, Ar**H**), 7.64-7.62 (d, *J* = 8.0 Hz, 2H, Ar**H**), 7.40-7.39 (d, *J* = 7.6 Hz, 2H, Ar**H**). Elemental analysis calcd (%) for $C_{13}H_6Br_2O$ (338.00): C, 46.20; H, 1.79; found: C, 46.45; H, 1.91.

Compound **4**:

To a previously degassed 1,4-dioxane (25 mL) solution of 2,7-dibromo-9-fluorenone (300 mg, 0.89 mmol) were added bis(pinacolato)diboron (580 mg, 2.28 mmol), $PdCl_2$(dppf) (40 mg), and KOAc (420 mg, 4.28 mmol), and the mixture was stirred at 100 °C overnight. After the solution was cooled, the dioxane was removed under vacuum, and then CH_2Cl_2 and water were added. The resulting mixture was extracted with dichloromethane (100 mL) twice, and the organic layer was washed with water and brine and then dried over $MgSO_4$. The organic solvent was concentrated in vacuo to yield a dark-black solid. The pure Compound **4** was isolated by silica gel column chromatography (using as eluents 1:15 ethyl acetate/ petroleum ether) as yellow solid. Yield: 200 mg (52.2%). ^{1}H-NMR (400 MHz, $CDCl_3$), *δ* (ppm): 8.13 (s, 2H, Ar**H**), 7.96-7.94 (d, *J* = 7.4 Hz, 2H, Ar**H**), 7.57-7.55 (d, *J* = 7.4 Hz, 2H, Ar**H**), 1.35 (s, 24H, 8C**H**$_3$). Elemental analysis calcd (%) for $C_{25}H_{30}B_2O_5$ (432.13): C, 69.49; H, 7.00; found: C, 69.71; H, 7.19.

Compound **5**:

Compound **5** was synthesized according to literature procedures in ref 12.

Compound **6**:

NBS powder (90 mg, 0.51 mmol) was added stepwise to a stirred solution of compound **5** (500 mg, 0.51 mmol) dissolved in dry THF (20 mL) at 0 °C in dark. The mixture was stirred overnight, quenched with water and then was extracted with dichloromethane (3 ×30 mL). The combined

organic phase was dried by anhydrous Na_2SO_4.The solvent was evaporated under reduced pressure and the reaction mixture was purified by column chromatography on silica gel using petroleum ether: ethyl acetate (150: 1) as the eluent to afford compound **6** as a white solid. Yield: 320 mg (59.3%). ^{1}H-NMR (300MHz, $CDCl_3$), δ (ppm): 7.45-7.42 (d, J = 8.8 Hz, 2H, Ph**H**), 7.00-6.93 (m, 4H, 2Ph**H**, 2Thiophene**H**), 6.62 (s, 2H, Ph**H**), 4.97 (s, 2H, OC**H**$_2$), 4.00-3.93 (m, 6H, 3OC**H**$_2$), 1.84-1.70 (m, 6H, 3OCH$_2$C**H**$_2$), 1.47 (m, 6H, 3OCH$_2$CH$_2$C**H**$_2$), 1.26 (m, 72H, 36C**H**$_2$), 0.91-0.86 (t, J = 6.6 Hz, 9H, 3C**H**$_3$). Elemental analysis calcd (%) for $C_{65}H_{109}BrO_4S$ (1066.55): C, 73.20; H, 10.30; found: C, 73.37; H, 10.52.

Compound FO16:

A mixture of Compound **4** (22 mg, 0.05 mmol), Compound **6** (160 mg, 0.15 mmol), K_2CO_3 (83 mg, 0.60 mmol), $Pd(PPh_3)_4$ (5 mg), THF (8 mL) and H_2O (8 mL) was refluxed at 78 °C for 36 h under an argon atmosphere. After the reaction was complete (TLC), the mixture was cooled to RT, and then the reaction mixture was extracted with dichloromethane (3 × 50 mL). The combined organic layer was dried with anhydrous Na_2SO_4, and the solvent was evaporated in *vacuo*. The residue was purified by a silicagel column chromatography (petroleum ether/ dichloromethane = 1 : 1) to produce compound FO16 as red solid. Yield: 75 mg (68.5%). ^{1}H-NMR (300MHz, $CDCl_3$), δ (ppm): 7.93 (s, 2H, Ph**H**), 7.75-7.72 (m, 2H, Ph**H**), 7.58-7.55 (d, J = 8.7 Hz, 4H, Ph**H**), 7.54-7.51 (d, J = 7.8 Hz, 2H, Ph**H**), 7.36-7.34 (d, J = 3.6 Hz, 2H, Thiophene**H**), 7.21-7.20 (d, J = 3.9 Hz, 2H, Thiophene**H**), 7.02-7.00 (d, J = 8.7 Hz, 4H, Ph**H**), 6.63 (s, 4H, Ph**H**), 4.99 (s, 4H, 2OC**H**$_2$), 4.01-3.93 (m, 12H, 6OC**H**$_2$), 1.84-1.70 (m, 12H, 6OCH$_2$C**H**$_2$), 1.53-1.42 (m, 12H, 6OCH$_2$CH$_2$C**H**$_2$), 1.35-1.22 (m, 144H, 72C**H**$_2$), 0.90-0.85 (t, J = 6.3 Hz, 18H, 6C**H**$_3$). ^{13}C-NMR (100MHz, $CDCl_3$): δ = 193.44, 158.85, 153.52, 144.44, 142.74, 141.36, 138.30, 135.48, 135.29, 131.79, 131.30, 127.30, 127.09, 124.77, 123.30, 121.21, 120.87, 115.51, 106.37, 73.60, 70.71, 69.37, 32.09, 30.52-29.52 (multi carbons in alkyl chain), 26.31, 26.28, 22.84, 14.25. MALDI-TOF: *m/z* calculated for $C_{143}H_{224}O_9S_2Na^+$, $[M+Na]^+$: 2172.640, found 2172.644. Elemental analysis calcd (%) for $C_{143}H_{224}O_9S_2$ (2151.48): C, 79.83; H, 10.49; found: C, 79.41; H, 10.18.

Compound FCN16:

Malononitrile (9.2 mg, 0.14 mmol) and compound FO16 (30 mg, 0.014 mmol) were dissolved in dry pyridine (10 mL) and the solution was stirred at 20 °C for 1 h to obtain a brown suspension. Pyridine (10 mL) was added and the mixture was stirred for an additional 5 h, followed by heating to 80 °C for 1 h. Ethanol (20 mL) was added to the solution and the mixture was cooled to 20 °C. A green solid of FCN16 was obtained after suction filtration and washed by acetonitrile. Yield: 18 mg (58.7%). ^{1}H-NMR (300MHz, $CDCl_3$), δ (ppm): 8.53 (s, 2H, Ph**H**), 7.67-7.64 (m, 2H, Ph**H**), 7.53-7.50 (d, J = 8.7 Hz, 4H, Ph**H**), 7.49-7.46 (d, J = 8.1 Hz, 2H, Ph**H**), 7.28-7.27 (d, J = 3.6 Hz, 2H, Thiophene**H**), 7.13-7.12 (d, J = 3.9 Hz, 2H, Thiophene**H**), 6.98-6.95 (d, J = 8.7 Hz, 4H, Ph**H**), 6.62 (s, 4H, Ph**H**), 4.95 (s, 4H, 2OC**H**$_2$), 4.00-3.93 (m, 12H, 6OC**H**$_2$), 1.84-1.70 (m, 12H, 6OCH$_2$C**H**$_2$), 1.52-1.42 (m, 12H, 6OCH$_2$CH$_2$C**H**$_2$), 1.35-1.24 (m, 144H, 72C**H**$_2$), 0.90-0.86 (t, J = 6.3 Hz, 18H, 6C**H**$_3$). ^{13}C-NMR (100MHz, $CDCl_3$): δ = 161.15, 158.87, 153.51, 144.81, 140.59, 140.48, 138.21, 135.45, 135.18, 131.77, 131.00, 127.08, 125.10, 123.32, 121.11, 115.42, 106.28, 73.60, 70.64, 69.33, 32.09, 30.53-29.53 (multi carbons in alkyl chain), 26.29, 22.84, 14.26. MALDI-TOF: *m/z* calculated for $C_{146}H_{224}N_2O_8S_2Na^+$, $[M+Na]^+$: 2220.652, found 2220.651. Elemental analysis calcd (%) for $C_{146}H_{224}N_2O_8S_2$ (2199.52): C, 79.73; H, 10.27; found: C, 79.55; H, 10.06.

3. ^{1}H and ^{13}C NMR spectra for representative compounds

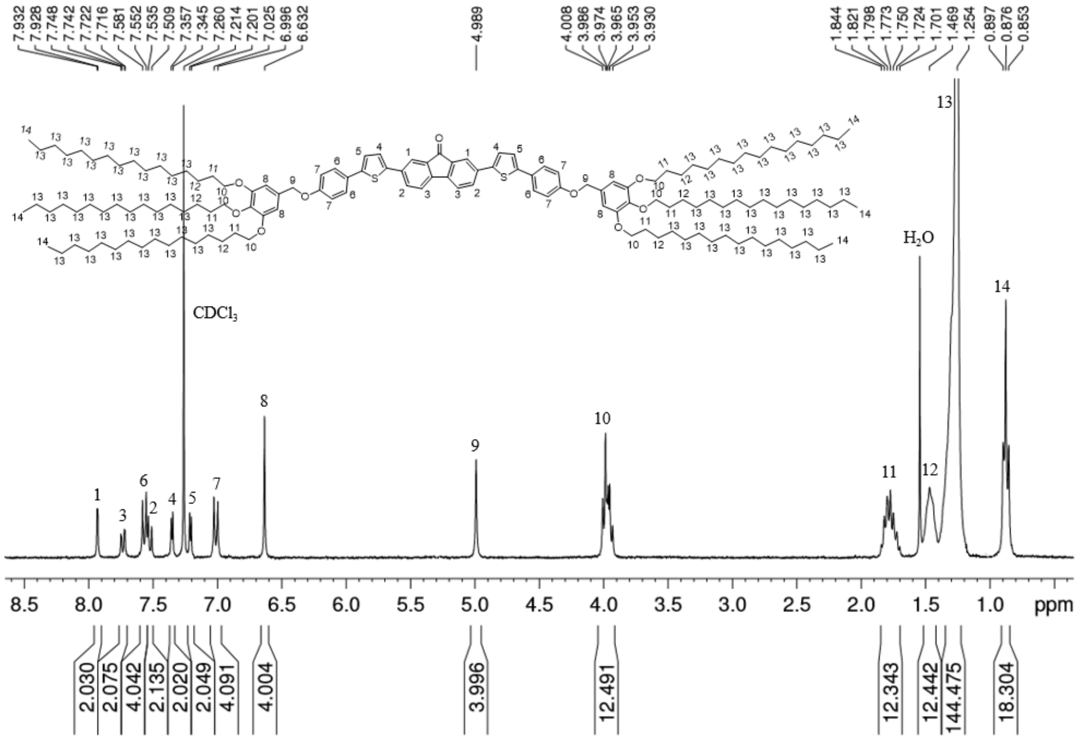

Supplementary Figure 26. ^{1}H NMR ($CDCl_3$, 400 MHz, ppm) spectra of compound FO16.

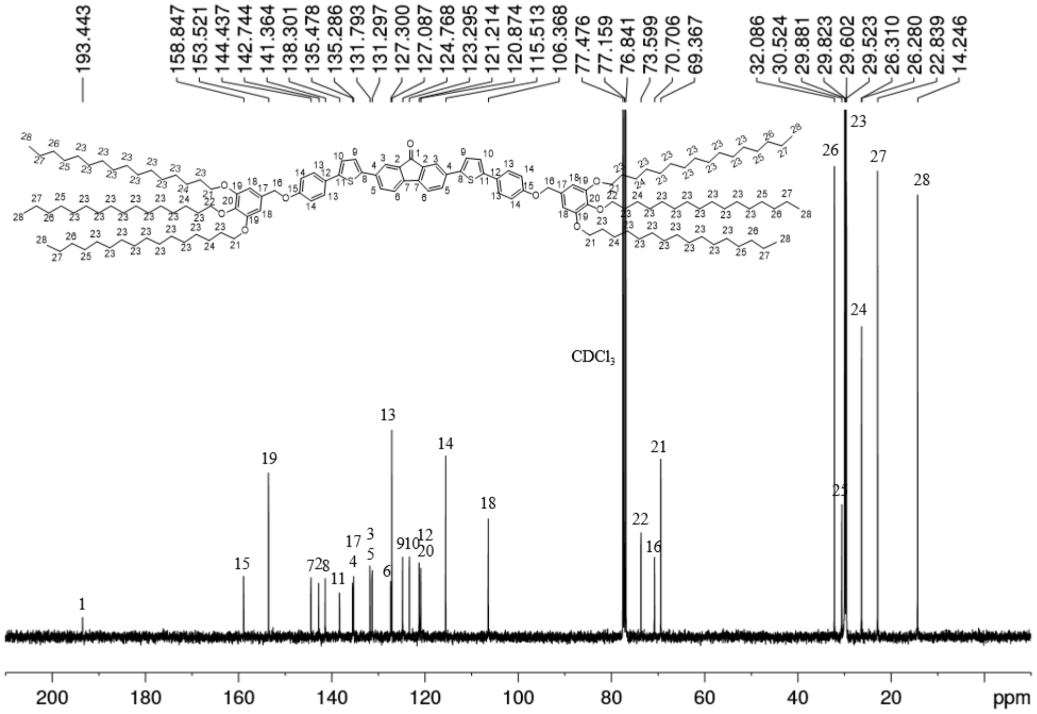

Supplementary Figure 27. ^{13}C NMR ($CDCl_3$, 100 MHz, ppm) spectra of compound FO16.

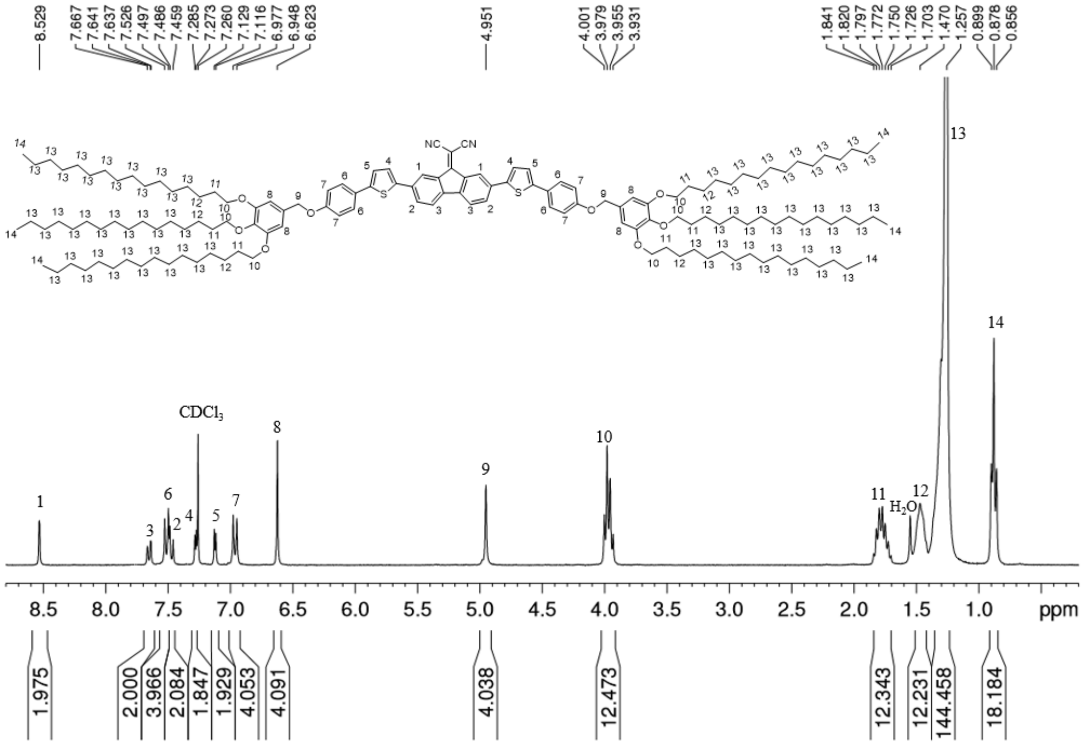

Supplementary Figure 28. ^{1}H NMR ($CDCl_3$, 400 MHz, ppm) spectra of compound FCN16**.**

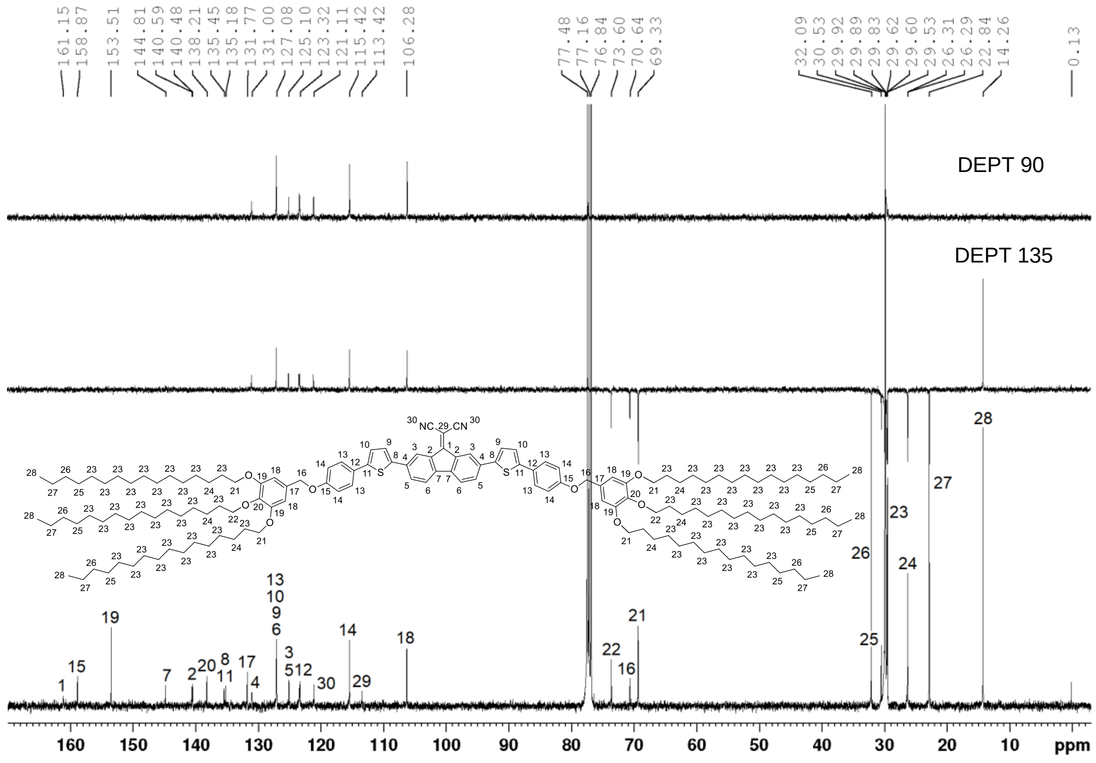

Supplementary Figure 29. ^{13}C NMR ($CDCl_3$, 100 MHz, ppm) spectra of compound FCN16**.**

4. MALDI-TOF-MS spectra of FO16 and FCN16

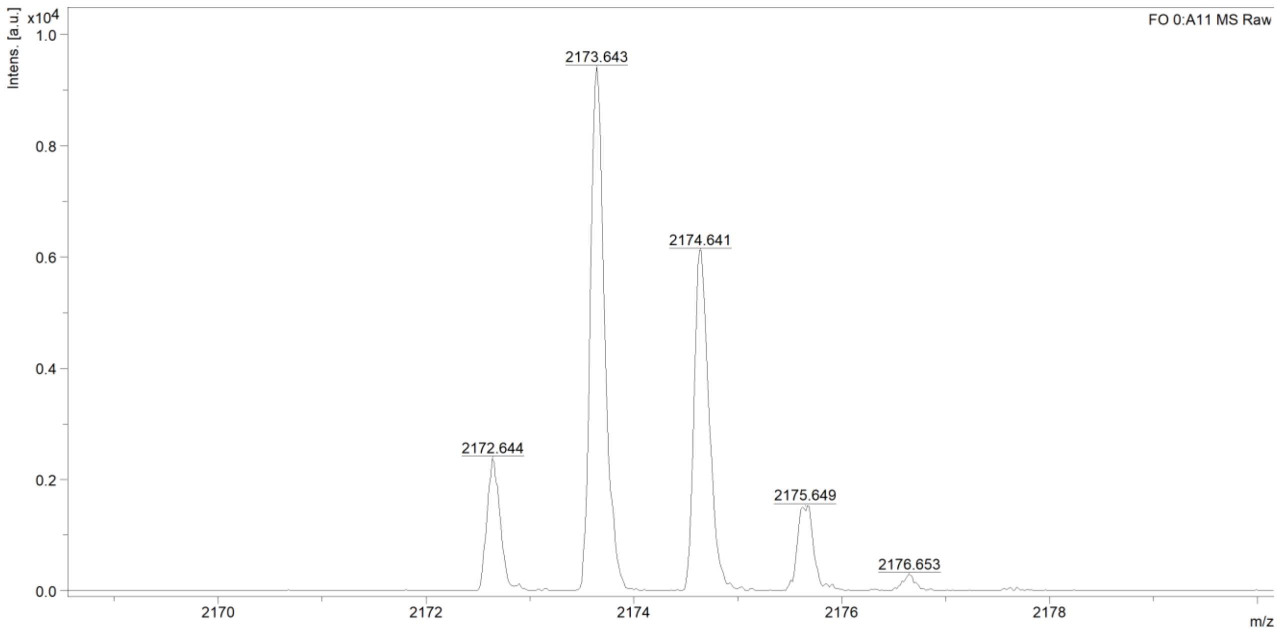

Supplementary Figure 30. The MALDI-TOF-MS spectra of FO16. (MALDI-TOF: *m*/*z* calculated for $C_{143}H_{224}O_9S_2Na^+$, $[M+Na]^+$: 2172.640, found 2172.644.

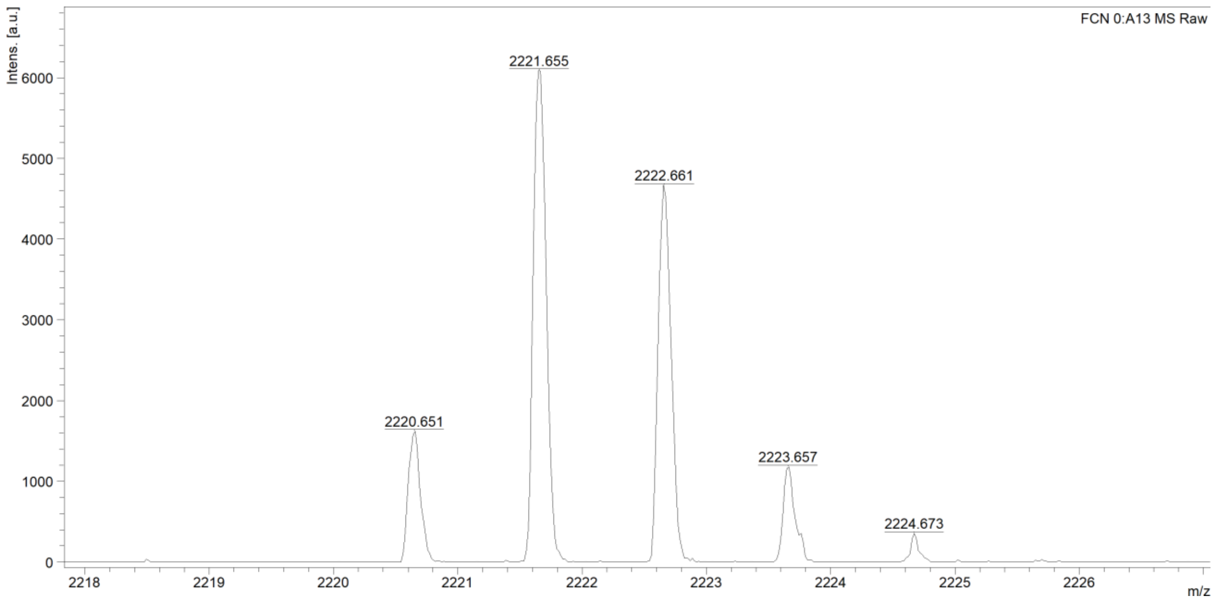

Supplementary Figure 31. The MALDI-TOF-MS spectra of FCN16. (MALDI-TOF: *m/z* calculated for $C_{146}H_{224}N_2O_8S_2Na^+$, $[M+Na]^+$: 2220.652, found 2220.651.)

Supplementary References

1. Cheng H. F. *et al.* Trigonal columnar self-assembly of bent phasmid mesogens. *Chem. Commun.* **54**, 156-159, (2018).
2. Immirzi A. & Perini B., Prediction of density in organic crystals, *Acta Crystallogr. A* **33**, 216-218, (1977).
3. Tyler C. A. & Morse D. C. Orthorhombic Fddd network in triblock and diblock copolymer melts. *Phys. Rev. Lett.* **94**, 208-302, (2005).
4. Tauc J., Grigorovici R. & Vancu A. Optical Properties and Electronic Structure of Amorphous Germanium. *physica status solidi (b)* **15**, 627-637, (1966).
5. Hosticka C., Bose T. K. & Sochanski J. S., Generalized treatment of the quadrupole-quadrupole interaction of low symmetry molecules and its effect on the second dielectric virial coefficient, *J. Chem. Phys.* **61**, 2575-2579, (1974).
6. Fontes E., Heiney P. A. & de Jeu W. H. Liquid-Crystalline and Helical Order In a Discotic Mesophase. *Phys. Rev. Lett.* **61**, 1202-1205, (1988).
7. Ungar G., Percec V., Holerca M. N., Johansson G. & Heck J. A. Heat-shrinking spherical and columnar self-assemblies, their interconversion and dependence on molecular taper angle, *Chem. Eur. J.* **6**, 1258-1266 (2000).
8. Yen M. H., Chaiprapa J, Zeng X. B., Liu Y. S., Cseh L, Mehl G. M. & Ungar G. Added Alkane Allows Thermal Thinning of Supramolecular Columns by Forming Superlattice - An X-ray and Neutron Study *J. Am. Chem. Soc.*, **138**, 5757–5760 (2016).
9. Fall W. S., Yen M. H., Zeng X. B., Cseh L., Liu Y.-S., Gehring G. A. & G. Ungar "Quantised Molecular Ejection Transition in Liquid Crystal Columns Self-Assembled from Wedge-Shaped Minidendrons", *Soft Matter.*, **15**, 22–29 (2019).
10. Zhao K. *et al.* Control the self-assembly of fluorenone-based polycatenars by tuning chain length. *Tetrahedron* **75**, 409-415, (2019).
11. Lee T. *et al.* Synthesis, Structural Characterization, and Unusual Field-Effect Behavior of Organic Transistor Semiconductor Oligomers: Inferiority of Oxadiazole Compared with Other Electron-Withdrawing Subunits. *J. Am. Chem. Soc.* **131**, 1692-1705, (2009).
12. Fang H. *et al.* Benzothiadiazole-based D-π-A-π-D fluorophores: Synthesis, self-assembly, thermal and photophysical characterization. *Dyes and Pigments* **147**, 190-198, (2017).
