# Supplementary material for: A case of antiferrochirality in a liquid crystal phase of counter-rotating staircases
Source: Nat Commun. 2022 Jan 19;13:384. doi: 10.1038/s41467-022-28024-1 (PMC8770800; doi:10.1038/s41467-022-28024-1)
Supplement: Supplementary file 2 — Description of Additional Supplementary Files [file 41467_2022_28024_MOESM2_ESM.pdf]

Description of Additional Supplementary Files

File Name: Supplementary Movie 1
Description: 3D electron density map of a unit cell of IC3 /12 in Fddd phase.

File Name: Supplementary Movie 2
Description: IC3/12: stylized 3D model of molecules in a Fddd unit cel; blue rods are aromatic molecular cores.

File Name: Supplementary Movie 3
Description: Compound IC3 /12: Helical rotation of column cross-sections with increasing z-elevation in Fddd phase; left: ED map (blue = high density aromatic regions), right: stylized model of arrangement of molecular cores.

File Name: Supplementary Movie 4
Description: 3D electron density map of FCN16 unit cell in Fddd phase.

File Name: Supplementary Movie 5
Description: FCN16: stylized 3D model of molecules in a Fddd unit cell.

File Name: Supplementary Movie 6
Description: Compound FCN16: Helical rotation of column cross-sections with increasing z-elevation in Fddd phase; left: ED map, right: stylized model.
